# Wine Polyphenol Content and Its Influence on Wine Quality and Properties: A Review

**DOI:** 10.3390/molecules26030718

**Published:** 2021-01-30

**Authors:** Rocío Gutiérrez-Escobar, María José Aliaño-González, Emma Cantos-Villar

**Affiliations:** IFAPA Rancho de la Merced, Consejería de Agricultura, Ganadería, Pesca y Desarrollo Sostenible, Junta de Andalucía, Cañada de la Loba, 11471 Jerez de la Frontera, Cádiz, Spain; rocio.gutierrez@juntadeandalucia.es (R.G.-E.); emma.cantos@juntadeandalucia.es (E.C.-V.)

**Keywords:** phenolic compounds, anthocyanidins, flavonols, flavanols, wine, quality, organoleptic properties, health benefits, extraction, winemaking technologies

## Abstract

Wine is one of the most consumed beverages around the world. It is composed of alcohols, sugars, acids, minerals, proteins and other compounds, such as organic acids and volatile and phenolic compounds (also called polyphenols). Polyphenols have been shown to be highly related to both (i) wine quality (color, flavor, and taste) and (ii) health-promoting properties (antioxidant and cardioprotective among others). Polyphenols can be grouped into two big families: (i) Flavonoids, including anthocyanidins, flavonols, flavanols, hydrolysable and condensed tannins, flavanones, flavones and chalcones; and (ii) Non-flavonoids, including hydroxycinnamic acids, hydroxybenzoic acids, stilbenes, tyrosol and hydroxytyrosol. Each group affects in some way the different properties of wine to a greater or a lesser extent. For that reason, the phenolic composition can be managed to obtain singular wines with specific, desirable characteristics. The current review presents a summary of the ways in which the phenolic composition of wine can be modulated, including (a) invariable factors such as variety, field management or climatic conditions; (b) pre-fermentative strategies such as maceration, thermovinification and pulsed electric field; (c) fermentative strategies such as the use of different yeasts and bacteria; and (d) post-fermentative strategies such as maceration, fining agents and aging. Finally, the different extraction methods and analytical techniques used for polyphenol detection and quantification have been also reviewed.

## 1. Introduction

Wine is one of the oldest and most widespread alcoholic beverages consumed in the world. In 2019, the world wine consumption was 246 Mill. hL according to the latest data collected by the IOV (International Organization of Vine and wine) [1]. The United States, France, Italy, Germany and China are the main consumers, with an average of 120 million hectoliters consumed per year. The pleasure of tasting wine and the cultural tradition in some countries are some of the reasons that encourage consumers to drink it in preference to other alcoholic beverages.

Numerous researchers have proved that moderate consumption of wine is beneficial to health since it presents protective effects against neurological diseases, cancer, diabetes, and cardiovascular diseases [2,3]. In general, wine is composed of alcohol, sugars, acids, tannins, minerals, proteins, and other compounds, such as organic acids, volatile compounds, and phenolic compounds [4]. Polyphenols have been reported to have antioxidant, anti-aging, anti-inflammation, anti-obesity, cardioprotective, neuroprotective, antibacterial, antiviral, antifungal, antiproliferative, anti-inflammatory, anti-allergic, anti-hypertensive and antithrombotic properties, and positive effects on human microbiota composition and functionality [3,5,6,7]. However, the health effects of polyphenols depend on both the amount consumed and their bioavailability. In comparison with other alcohols, these healthy properties endow wine with added value, largely associated with polyphenols (mainly resveratrol). These bioactive properties of wine were clearly established in the conclusions of the so-called “French paradox”.

The “French paradox” concept was formulated in the 1980s by French epidemiologists [8], who compared the mortality rate from cardiovascular disease and the cholesterol level in different populations of the world. This correlation was positive in all the cities studied, except in Toulouse, which showed the same cholesterol levels as Glasgow but with a lower mortality rate from cardiovascular disease [8]. The discrepancy between the risk of cardiovascular disease (high blood cholesterol) and death as a result of it was called the “French paradox”. In 1992, the French authors Renauld and De Lorgeril explained this paradox by the consumption of the Mediterranean diet, with an abundance of vegetables, fruits, olive oil and especially red wine [9]. After an in-depth study of the variables that could contribute to this discrepancy, the researchers concluded that moderate wine consumption improved cardiovascular outcomes despite a high-fat diet [8,9,10].

Additionally, a study carried out in France and Denmark showed that the moderate consumption of wine caused a decrease in mortality (24–31%) from cardiovascular disease compared to the equivalent consumption of beer or spirits [11]. It has also been described that moderate wine consumption may ensure people a longer life expectancy than both those consuming wine in excess and those who drink none at all [12,13]. 

Consumer demands and the highly competitive market force wineries to produce high quality wines. The quality of a wine is determined by the interaction of physicochemical properties such as alcoholic strength, residual sugar content, density, total acidity, volatile acidity and sulfites together with sensory properties such as aroma, flavor, astringency, bitterness, color, turbidity, or unpleasant odors. These parameters depend on intrinsic factors such as the grape variety and extrinsic ones including soil, weather and winemaking techniques. The interaction of all of these factors determines a profile of compounds directly involved in the quality of the wines [14]. 

Phenolic compounds are a key factor in the quality of wines, especially red ones. A better understanding of their extreme complexity will allow us to consider their use as a quality criterion. The following sections present a review of the different polyphenols, highlighting their influence on the quality of the wine. To the best of the author’s knowledge, the most important factors that affect their content in grapes and wines and the most actual researcher about this topic are described. In addition, the extraction methods and the most frequently used analysis techniques for their determination in wines are also reviewed. Some of the advantages and disadvantages associated with both of them have been included with the aim of easing future researcher’s decisions for the polyphenol analysis. Conclusion includes some recommendations for the management of internal and external factors depending the type of wine to elaborate. 

## 2. Polyphenols: Key Compounds for Wine Quality

Phenolic compounds are natural substances that are composed of one or more hydroxyl groups attached to one or more aromatic or benzene rings. Polyphenols can be found in many vegetables and fruits, including grapes, and therefore in must and wine. However, as discussed below (Section 3), their content is greatly influenced by the type of grape used, the technological practices to which grapes are exposed [4], the type of yeast used in the alcoholic fermentation, and the contact with solid parts of the grape during the maceration [15]. It is important to remember that red wines are exposed to all grape parts during the winemaking process. For this reason, their polyphenol concentration is higher (1–5 g/L) than in white wines (0.2–0.5 g/L), the contents of which are essentially originated from the pulp, as the types and proportions of polyphenols are different in the pulp, skin and seeds of grapes [5]. Rose wines present an intermediate polyphenol content, with values between those for red and white wines [15,16]. The polyphenols in wine determine many of its sensory properties such as appearance, color, astringency, bitterness, and flavor [17,18,19], and also its stability through subsequent oxidative processes (browning in white wines and oxidation in red wines).

It has been considered that polyphenol compounds have a significant influence on aroma compounds due to they are usually associated with volatile compounds through intermolecular interactions with important consequences on the loss of aroma [20,21,22]. It is for example the case of malvidin that can be bonded to acetosyringone, syringaldehyde, acetovanillone, vanillin, 3,5-dimethoxyphenol, and 4-ethylguaiaco. Or catechin, caffeic acid, and quercetin, which are commonly bonded to aroma compounds such as isobutyl methoxypyrazine, 3-mercaptohexanol, 3-mercaptohexanol acetate, and ethyl decanoate. However, a deep study about the influence of these unions in the aroma and sensory properties has not been carried out. In addition, they contribute to the health-promoting properties of wine mentioned in the previous section [23]. 

According to their chemical structure, polyphenols present remarkably diverse structures, from simple phenolic acids to high molecular mass polymeric forms such as hydrolysable and condensed tannins, respectively.

They are usually found in conjugated forms with sugar residues by *β*-glycosidic bonds (*O*-glycosylated) or by direct bonds of the sugar to a carbon atom of the aromatic ring (C-glycosides) [5,23]. Glucose is the main sugar in fruit skins, which is why many phenolic compounds are bonded to it. However, they can also be found bonded to galactose, rhamnose, xylose, arabinose, as well as glucuronide, galacturonic and other acids [23]. Another factor that could modify the phenolic compounds’ nature and that should be taken into account is malolactic fermentation. Malolactic fermentation is catalyzed by bacteria acid lactic that decarboxylates malic acid to lactic acid, which results in deacidification, which affects polyphenolic compounds in a different manner depending on their structure. It is, for example, the case of anthocyanins that turned into their colorless form during this process due to the changes in pH and the increment in acidity [24]. Or some glucosides can suffer hydrolysis reactions for this change in pH [25]. Moreover, malolactic fermentation provides microbiological stability and improves the final aroma balance by modifying fruit-derived aromas and producing aroma-active compounds [26,27,28].

It is possible to distinguish an extensive family of phenolic compounds, which are grouped into flavonoids and non-flavonoids.

**Figure 1:**
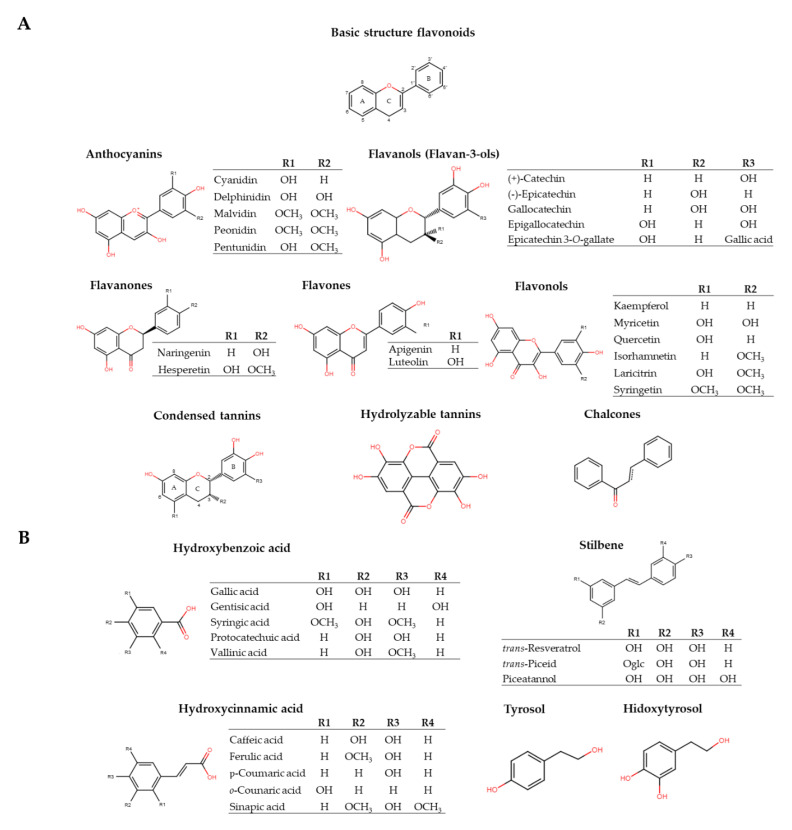
(**a**) Structure of the flavonoid compounds in wine; (**b**) Structure of the non-flavonoid compounds in wine. (Own elaboration).

- **Flavonoids**: flavonoids are made up a C_15_ (C_6_-C_3_-C_6_) type structure (benzene) linked by a 3-carbon chain cyclized through oxygen (Figure 1a). This carbon skeleton and the multiple radicals bonded to it are responsible for the chemical diversity of this family [4]. All the flavonoids found in grapes and wine have a hydroxyl group in position 5 and 7 of the A ring [29]. The antioxidant action of flavonoids depends mainly on their ability to reduce free radicals and chelate metals (Cu and Zn), preventing the catalytic reactions of free radicals [30]. This family comprises anthocyanidins, flavanols, flavonols, flavanones, flavones, chalcones and tannins (condensed tannins and hidrolysable tannins).
  ○ *Anthocyanidins* are natural, water-soluble pigments responsible for the red color of grapes and red wines. Anthocyanin pigments are mainly composed of aglycones (anthocyanidins) bonded sugar (anthocyanins) (Figure 1a). Five anthocyanidins have been identified in both grapes and wine: delphinin, cyanidin, petunidin, peonidin and malvidin. The color of the anthocyanin changes depending on the pH, the concentration of sulfur dioxide, and the copigments present in the wine. At a low pH (less than 4), all anthocyanidins are in the flavan (red) cation form. When the pH increases, the intensity of color increases, from colorless to violet or blue in alkaline or neutral solutions [24]. The anthocyanin concentration can range between 90 and 400 mg/L [29], to concentrations above 700 mg/L in aged red wine, whereas in white wine they are absent [5]. When anthocyanidins interact with other phenolic compounds in wine, a phenomenon known as co-pigmentation occurs, which usually stabilizes the anthocyanidins, and therefore the color [31].
  ○ *Flavanols* (flavan-3-ols) are found in monomeric form (catechin and epicatechin) and in their polymeric form (proanthocyanidins, also called condensed or non-hydrolysable tannins). The following flavan-3-ols are the main ones found in the skin and seed of grapes: (+) catechin, (−) epicatechin, epigallocatechin and epicatechin 3-*O*-gallate (Figure 1a) [32,33]. Flavanols are responsible for the stabilization of both the color and sensory characteristics (mainly astringency and bitterness) of wines [34]. The concentration range detected in young white wine is from 15 to 25 mg/L, and from 4 to 120 mg/L in young red wine [5,35].
  ○ *Flavonols* [4,5] are yellow pigments found in the skin of grapes characterized by a double bond between C_2_ and C_3_ and by the presence of a hydroxyl group in position 1 (Figure 1a). They are usually present in glycosidic forms, linked to a sugar (glucose or rhamnose), but others such as galactose, arabinose, xylose or glucuronic acid may also be involved. The main flavonols described in grapes and wine are myricetin, quercetin, laricitrin, kaempferol, isorhamnetin and syringetin [36]. Flavonols are present in both white and red wines. In white wines the proportion that affects the color is exceedingly small, while in red wines the yellow color is masked by the purplish red of the anthocyanidins [37]. Furthermore, the flavonoid color can change from white to yellow and they consequently play an important role in the color stabilization of young red wines, through the copigmentation interaction with anthocyanidins [38]. In addition, they have an important role in the sensory perception of astringency and bitterness [24]. In red wine, the maximum content described is 60 mg/L [29,39].
  ○ *Condensed tannins* are the result of the condensation of flavanols (flavan-3-ols) (Figure 1a). Epicatechin is the most abundant condensed tannin in grapes and wine, followed by catechin. B-type proanthocyanidins, and in particular dimers B1, B2 and B4 or trimer procyanidin C1, are mainly located in grape skins and seeds [40]. These tannins increase during the aging of the wine and can form insoluble polymers [41], increasing astringency with tannin concentration. Natural condensed tannins can be found at concentrations levels from 1.2 to 3.3 g/L [42,43].
  ○ *Flavanones* have a saturated carbon chain between the C_2_ and C_3_ atoms, often named dihydroflavones by analogy with the flavones [4]. Naringenin is the main compound in wine, reaching 25 mg/kg in reds and 7.7 mg/kg in whites [5].
  ○ *Flavones* are characterized by the presence of a double bond between carbons C_2_ and C_3_ and by the absence of a hydroxyl group in the C_3_ position. Isoflavones are isomers of flavones, displaying the aromatic ring B in the C_3_ position [4]. Flavones can be present in wine in levels ranging from 0.2 to 1 mg/L [44,45].
  ○ *Chalcones* are a subclass of flavonoids with two aromatic rings linked by a carbonylic *α, β*-unsaturated system (Figure 1a). Chalcone derivatives are important intermediates and are precursors for a vast range of flavonoid derivatives found in grapes or wine [4].
  ○ *Hydrolysable tannins* are high molecular weight molecules, composed mainly of esters of gallic acid (gallotannins) and ellagic (ellagitannins) bonded to glucose or other sugars (Figure 1a). They are more susceptible to hydrolysis than condensed tannins induced by pH changes, enzymatic or non-enzymatic processes [46]. The hydrolysable tannins are not found in *Vitis vinifera*, only in grapes of the muscadine subgenus and in wines aging in barrels and are thus proposed in the literature as a marker of maturity. The final content of hydrolysable tannins can vary widely, from 0.4 to 50 mg/L [43,47,48].
- **Non-flavonoids** form an extensive family within polyphenols, generally having a simpler structure than that of flavonoids. They are mainly composed of phenolic acids (hydroxybenzoic acids and hydroxycinnamic), and stilbenes [4,5]. These groups can reach a concentration range from 60 to 566 mg/L in red wine [46].
  ○ *Hydroxybenzoic acids* have a C_6_-C_1_ structure derived from benzoic acid (Figure 1b). The most abundant are *p*-hydroxybenzoic, gallic, vanillic, gentisic, syringic, salicylic, and protocatechuic acids [49]. The total amount of hydroxybenzoic acids in red wine is expected to range from undetectable to 218 mg/L [46]. Gallic acid is considered the most important phenolic acid in red wine with a concentration of around 70 mg/L, while levels can reach 10 mg/L in white wine [29]. It stands out for being the precursor of all hydrolysable tannins.
  ○ *Hydroxycinnamic acids* have a C_6_-C_3_ structure, are very abundant, diverse and all come from cinnamic acid (Figure 1b). The main examples are caffeic, coumaric, sinapic and ferulic acids, essentially conjugated with tartaric acid esters or diesters [50]. Hydroxycinnamic acids are the third most abundant group of polyphenols in grapes and the predominant group in must and white wine. They are easily oxidizable and are associated with wine browning processes. They also are precursors of volatile phenolic compounds [51]. The average amount of hydroxycinnamic acids quantified is about 100 and 30 mg/L in red and white wines, respectively [5] although some authors have found higher concentrations: 130 mg/L in white wines and 60 mg/L in red wines [29].
  ○ *Stilbenes* are bioactive compounds consisting of two aromatic rings linked by ethyl positions (Figure 1b). The main sources of stilbenes in the human diet are grapes and their derivatives: juice and wine [52]. The main stilbenes described in *Vitis vinifera* wines are *trans*-piceid and *trans*-resveratrol, with hopeaphenol, ampelosin A, isohopeaphenol, piceatannol, pallidol, ɛ-viniferin, miyabenol C, r-viniferin, r2-viniferin also being detected [53,54,55,56]. They are found naturally in wine, but at low concentrations (0–5 mg/L) [6]. However, when grapes are exposed to biotic or abiotic stress, the levels of resveratrol (the most studied compound), its glycoside called piceid, and its dimeric and trimeric forms (e.g., pallidol, viniferins) may range from negligible up to more than 100 mg/L [5]. Recently, some stilbenes have been quantified by UPLC-MS/MS, *trans*-piceid being the most abundant in white wine (average of 155 μg/L), and *cis*- and *trans*-piceids and hopeaphenol in red wine (average of 3.73 and 3.16 mg/L, respectively) (mean 1.55 mg/L) [57].
  ○ *Tyrosol* is a natural phenolic antioxidant compound found mainly in olive oil, although there are studies that have detected it in white and red wines (Figure 1b). Some results showed values up to 45 mg/L in white wine and between 20 and 60 mg/L in red wines [4].
  ○ *Hydroxytyrosol* (HT) (3,4-dihidroxifeniletanol) is a phenyl ethyl alcohol, mainly responsible for the antioxidant properties of olive oil (Figure 1b). In 2011, it was accepted as protective compound against oxidative damage [58]. It is found naturally in red wine at concentrations between 1.98 and 3.89 mg/L [6,59]. It seems to be synthesized during alcoholic fermentation by yeasts [60].

All the above polyphenols largely define the quality of wine, due to their contribution to its sensory properties: color, taste, mouthfeel, flavor, astringency, and bitterness [4,61]. The present review examines the many factors influencing the polyphenol concentration in wine, and the different extraction and analytical techniques employed for their quantification.

## 3. External Factors and Polyphenol Content

**Figure 2:**
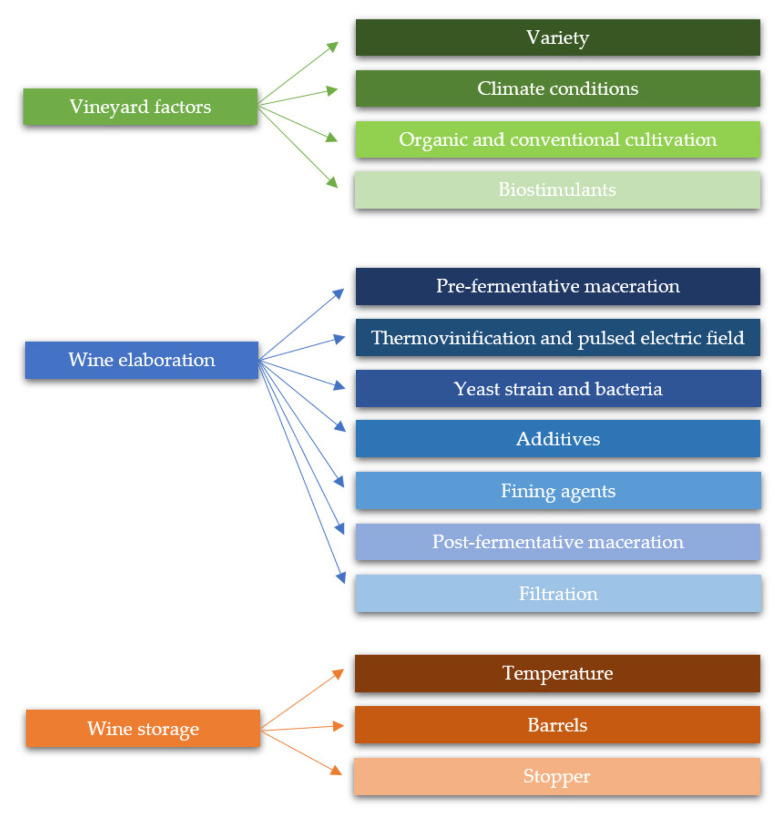
Summary diagram about the external factors selected to be studied and their influence on phenolic compounds in grape, must, and wine.

As previously mentioned, the phenolic content of wine has a decisive influence on its organoleptic properties, and therefore on its quality. Many internal and external factors have proved to significantly affect to the concentration of phenolic compounds. Some of these variables have been studied by researchers with the aim of controlling and/or modulating the phenolic concentration and the organoleptic properties associated with them in wine. It is possible to distinguish three decisive levels in which phenolic compounds may be modulated: (i) vineyard; (ii) winemaking; and (iii) wine storage. Below, an in-depth study of these levels and their influence on phenolic content is performed. A summary diagram can be found in Figure 2.

### 3.1. Vineyard Factors

The phenolic concentration of grapes is influenced by multiple factors. First, by the genetic factor, which implies that some varieties naturally produce higher concentrations of phenolic compounds than others. On the other hand, there are other factors related to vineyard management that have a direct effect on the phenolic compound concentration in grapes. Below, some of these factors, such as the climate conditions or terroir, biostimulants and early harvest, have been reviewed.

#### 3.1.1. Variety

Grape (*Vitis vinifera* L.) has proved to be one of the most abundant fruits worldwide with a high concentration of phenolic compounds. However, several authors have proved that the phenolic compound profile can change significantly according to the variety, genetics being one of the main factors to consider when wanting to achieve a high concentration of these compounds. In fact, the composition and content of the phenolic compounds may be used as markers to determine the variety of an unknown sample. This is, for example, the case of *Vitis Vinifera* hybrid Regent, which exhibits a high concentration of anthocyanin diglucosides, whereas most *Vitis Vinifera* varieties show a high content of anthocyanin monoglucosides [62,63]. Or varieties such as Tempranillo, which presents elevated malvidin content in comparison with other *Vitis Vinifera* varieties, a fact that could be easily used for the determination of this variety [64,65]. In general, red grapes exhibit a higher concentration of phenolic compounds than white grapes. However, a deeper study makes it possible to determine the specific profile of the different varieties.

Brezoiu et al. [66] studied the phenolic composition of two different red grape varieties (Feteasca Negra and Cabernet Sauvignon) by the solid-liquid extraction and HPLC-DAD methodology. The results are shown in Table 1. In this case, the number of phenolic compounds identified in grape extracts and their relative concentrations were considerably higher in the Cabernet Sauvignon variety. González de Peredo et al. [67] analyzed twelve varieties of *Vitis vinifera* grapes, quantifying the concentration of phenolic compounds by UHPLC (ultra-high pressure liquid chromatography). The results are shown in Table 1. As expected, the concentration of the 17 phenolic compounds (caffeic acid, caftaric acid, catechin, ECGc, epicatechin, gallic acid, kaempferol, myricetin, *trans*-piceid, procyanidin A2, procyanidin B1, procyanidin B2, protocatechuic acid, quercetin 3-*O*-galactoside, quercetin 3-*O*-glucoside, quercetin 3-*O*-rhamnoside, *trans*-resveratrol) was lower in the white grape varieties (Albariño, Chardonnay, and Gewurtztraminer) than in the red ones (Cabernet Sauvignon, Graciano, Malbec, Mencía, and Merlot). In contrast, white grapes exhibited a high degree of hydroxycinnamoyltartaric acids such as caftaric acid. 

Iacopini et al. [68] quantified phenolic compounds in seven varieties (Sangiovese, Merlot, Cabernet Sauvignon, Canaiolo Nero, Colorino del Valdarno, Foglia Tonda, and Montepulciano) cultivated in the Experimental Institute of Viticulture in Arezzo (Italy). Five phenolic compounds (catechin, epicatechin, resveratrol, rutin, and quercetin) were quantified by comparison of retention times with pure standards. The results can be found in Table 1. Montepulciano exhibited the highest catechin concentration and Canaiolo was the variety with the highest value of epicatechin, whereas Foglia Tonda showed the lowest content of catechin, epicatechin and quercetin. Cabernet Sauvignon manifested the highest values of resveratrol and Sangiovese showed the highest concentration of quercetin and rutin, and the lowest content of resveratrol. Finally, Colorino exhibited the lowest concentration of rutin.

Obreque-Slier et al. [69] analyzed two Carménère and Cabernet Sauvignon varieties from Chile with the aim of calculating their phenolic compound concentrations. A total of 15 phenolic compounds (caftaric acid, catechin, gallic acid, isorhamnetin 3-*O*-glucoside, kaempferol 3-*O*-galactoside, kaempferol 3-*O*-glucoside, malvidin 3-*O*-acetylglucoside, malvidin 3-*O*-coumarylglucoside, malvidin 3-*O*-glucoside, myricetin, procyanidin B3, quercetin 3-*O*-galactoside, quercetin 3-*O*-glucoside, syringic acid, vanillic acid) were identified but slight differences were observed in the content between both varieties, as shown in Table 1. Guerrero et al. [52] quantified 25 phenolic compounds (*trans*-caftaric acid, catechin, *trans*-coutaric acid, cyanidin 3-*O*-glucoside, cyanidin-3-*p*-coumaroylglucoside, delphinidin 3-*O*-acetylglucoside, delphinidin 3-*O*-glucoside, epicatechin, isorhamnetin 3-*O*-glucoside, kaempferol 3-*O*-glucoside, malvidin 3-*O*-acetylglucoside, malvidin-3-caffeoylglucoside, malvidin 3-*O*-glucoside, *cis*-malvidin-3-*p*-coumaroylglucoside, *trans*-malvidin-3-*p*-coumaroylglucoside, myricetin-3-*O*-glucuronide, myricetin-3-*O*-glucoside, peonidin 3-*O*-acetylglucoside, peonidin 3-*O*-glucoside, petunidin 3-*O*-acetylglucoside, petunidin-3-*p*-coumaroylglucoside, petunidin-3-*O*-glucoside, quercetin-3-*O*-rutinoside, syringetin-3-*O*-glucoside) from three autochthonous red grape varieties (Jaen Tinto, Palomino Negro, and Tintilla de Rota), one international variety (Cabernet Sauvignon) and one Spanish variety (Tempranillo). All of them were grown in a warm climate (Table 1). Tintilla de Rota exhibited the highest phenolic compound concentration followed by Cabernet Sauvignon. 

A comparison of the concentrations in each variety cannot be made since they were not cultivated under the same conditions and the extraction and analysis methodologies used were different.

**Table 1:** Concentration of phenolic compounds (mg/g) quantified in grape varieties analyzed under different conditions. * fw: fresh weight; dw: dry weight; ** ECGc: Epigallocatechin gallate; -: this compound has not been analyzed during this research.

**Variety**	**Albariño**	**Chardonnay**	**Gewurtztraminer**		**Cabernet Sauvignon**	**Graciano**	**Malbec**	**Mencía**	**Merlot**	**Syrah (Bahia)**	**Syrah (Pernambrusco)**		**Cabernet Sauvignon**	**Feteasca Negra**	
Compounds/Reference	González de Peredo et al. [67] (mg/g fw *)									de Oliveira et al. [70] (mg/g fw *)			Brezoiu et al. [66] (mg/g fw *)		
Caffeic acid	0.001	0.001	0.001		0.005	0.006	0.001	0.002	0.001	-	-		-	-	
Caftaric acid	0.357	0.443	0.267		0.406	0.161	0.426	0.482	0.249	-	-		-	-	
Catechin	0.02	0.014	0.087		0.026	0.033	0.02	0.022	0.047	-	-		0.184	-	
Cyanidin 3-*O*-glucoside	-	-	-		-	-	-	-	-	0.001	0.001		-	-	
Delphinidin 3-*O*-coumarylglucoside	-	-	-		-	-	-	-	-	0.001	-		-	-	
Delphinidin 3-*O*-glucoside	-	-	-		-	-	-	-	-	0.002	-		-	-	
ECGc **	0.016	-	0.107		0.015	0.153	0.168	0.051	0.137	-	-		-	-	
Epicatechin	0.008	0.008	0.009		0.009	0.008	0.008	0.008	0.07	-	-		2.666	2.27	
Gallic acid	0.006	0.002	0.003		0.008	0.003	0.007	0.004	0.005	-	-		1.171	1.069	
Isorhamenetin 3-*O*-Glucoside	-	-	-		-	-	-	-	-	-	-		-	-	
Kaempferol	0.192	0.191	0.193		0.193	0.214	0.194	0.198	0.189	-	-		-	-	
Myrcetin	-	-	-		-	-	-	-	-	-	-		0.172	0.055	
Peonidin 3-*O*-acetylglucoside	-	-	-		-	-	-	-	-	-	0.001		-	-	
Peonidin 3-*O*-coumarylglucoside	-	-	-		-	-	-	-	-	0.001	0.001		-	-	
Peonidin 3-*O*-glucoside	-	-	-		-	-	-	-	-	0.004	-		-	-	
Petunidin 3-*O*-coumarylglucoside	-	-	-		-	-	-	-	-	-	0.001		-	-	
Petunidin 3-*O*-glucoside	-	-	-		-	-	-	-	-	0.003	0.001		-	-	
*trans*-Piceid	0.048	0.006	0.012		0.017	0.017	0.014	0.03	0.017	-	-		-	-	
Procyanidin A2	0.07	0.001	0.004		0.083	0.228	0.016	0.118	0.012	-	-		-	-	
Procyanidin B1	0.271	0.136	0.134		0.365	0.494	0.207	0.397	0.496	-	-		-	-	
Procyaidin B2	0.035	0.013	0.019		0.03	0.056	0.016	0.047	0.059	-	-		-	-	
Protocatechuic acid	0.003	-	0.001		0.001	0.004	0.003	0.001	0.002	-	-		0.451	0.489	
Quercetin 3-*O*-galactoside	0.006	0.002	0.007		0.011	0.018	0.019	0.013	0.008	-	-		-	-	
Quercetin 3-*O*-Glucoside	0.004	0.002	0.006		0.005	0.012	0.016	0.008	0.003	-	-		-	-	
Quercetin 3-rhamnoside	-	-	-		-	-	-	0.002	-	-	-		-	-	
Rutin	-	-	-		-	-	-	-	-	-	-		0.532	-	
Syringic acid	-	-	-		-	-	-	-	-	-	-		2.031	1.917	
*trans*-Resveratrol	-	-	-		-	0.003	0.001	-	-	0.004	0.006		0.033	0.049	
Vanillic acid	-	-	-		-	-	-	-	-	-	-		0.368	1.088	
**Variety**	**Cabernet Sauvignon**	**Jaen Tinto**	**Palomino Negro**	**Tempranillo**	**Tintilla de Rota**	**Cabernet Sauvignon**	**Canaiolo Nero**	**Colorino del Valdarno**		**Flogia Tonda**	**Merlot**	**Montepulciano**	**Sangiovese**	**Cabernet Sauvignon**	**Carmènéere**
Compounds/Reference	Guerrero et al. [52] (mg/g fw *)					Iacopini et al. [68] (mg/g dw *)							Obreque-Slier et al. [69] (mg/g dw *)		
Caftaric acid	0.009	0.014	0.014	0.010	0.019	-	-	-		-	-	-	-	0.008	0.008
Catechin	0.004	0.003	0.003	0.006	0.002	1.418	1.407	1.244		0.674	1.388	2.057	0.969	0.051	0.031
*trans*-Coutaric acid	0.006	0.010	0.009	0.007	0.012	-	-	-		-	-	-	-	-	-
Cyanidin 3-*O*-glucoside	-	0.007	0.037	0.015	0.036	-	-	-		-	-	-	-	-	-
Cyanidin-3-*p*-coumaroylglucoside	-	-	0.019	-	0.014										
Delphinidin 3-*O*-acetylglucoside	0.021	-	0.018	0.01	-	-	-	-		-	-	-	-	-	-
Delphinidin 3-*O*-glucoside	0.046	0.028	0.197	0.107	0.098	-	-	-		-	-	-	-	-	-
Epicatechin	-	0.001	-	0.002	-	1.276	2.057	0.89		0.472	1.318	1.646	1.068	-	-
Gallic acid	-	-	-	-	-	-	-	-		-	-	-	-	0.033	0.034
Isorhamenetin 3-*O*-Glucoside	0.027	0.013	-	-	0.031	-	-	-		-	-	-	-	0.125	0.226
Kaemperol 3-*O*-Galactoside	-	-	-	-	-	-	-	-		-	-	-	-	0.083	0.124
Kaemperol 3-*O*-Glucoside	0.036	0.021	0.018	0.029	0.032	-	-	-		-	-	-	-	0.313	0.399
Malvidin 3-*O*-acetylglucoside	0.552	0.137	0.067	0.049	0.153	-	-	-		-	-	-	-	0.004	0.003
Malvidin-3-caffeoylglucoside	0.062	0.038	0.042	-	0.056	-	-	-		-	-	-	-	-	-
Malvidin 3-*O*-coumarylglucoside	-	-	-	-	-	-	-	-		-	-	-	-	0.002	0.003
Malvidin 3-*O*-glucoside	0.665	0.425	0.508	0.292	1.207	-	-	-		-	-	-	-	0.006	0.016
Malvidin-3-*p*-coumaroylglucoside (*cis*)	0.018	0.011	0.078	0.059	0.021	-	-	-		-	-	-	-	-	-
Malvidin-3-*p*-coumaroylglucoside (*trans*)	0.22	0.144	0.222	0.22	0.427	-	-	-		-	-	-	-	-	-
Myrcetin	-	-	-	-	-	-	-	-		-	-	-	-	0.104	0.151
Myricetin-3-*O*-glucuronide	0.05	0.052	0.06	0.032	0.131	-	-	-		-	-	-	-	-	-
Myricetin-3-*O*-glucoside	0.098	0.056	0.059	0.178	0.079	-	-	-		-	-	-	-	-	-
Peonidin 3-*O*-acetylglucoside	0.029	0.015	0.016	-	0.043	-	-	-		-	-	-	-	-	-
Peonidin 3-*O*-glucoside	0.043	0.043	0.096	0.03	0.437	-	-	-		-	-	-	-	-	-
Petunidin 3-*O*-acetylglucoside	0.026	-	0.027	0.015	-	-	-	-		-	-	-	-	-	-
Petunidin 3-*O*-glucoside	0.051	0.042	0.179	0.091	0.119	-	-	-		-	-	-	-	-	-
Petunidin-3-*p*-coumaroylglucoside	0.021	0.016	0.067	0.056	0.029	-	-	-		-	-	-	-	-	-
Procyaidin B3	-	-	-	-	-	-	-	-		-	-	-	-	0.037	0.035
Quercetin	-	-	-	-	-	0.006	-	0.005		0.003	0.007	0.008	0.011	-	-
Quercetin 3-*O*-Galactoside	-	-	-	-	-	-	-	-		-	-	-	-	0.19	0.181
Quercetin 3-*O*-Glucoside	0.07	0.032	0.041	0.115	0.103	-	-	-		-	-	-	-	1.095	1.812
Quercetin-3-*O*-rutinoside	0.069	0.035	0.043	0.133	0.100	-	-	-		-	-	-	-	-	-
Rutin	-	-	-	-	-	0.886	0.413	0.403		0.604	0.899	0.532	1.491	-	-
Syringetin-3-*O*-glucoside	0.067	0.023	-	-	0.062	-	-	-		-	-	-	-	-	
Syringic acid	-	-	-	-	-	-	-	-		-	-	-	-	0.015	0.031
*trans*-Caftaric acid	0.001	0.001	0.001	0.001	0.002	-	-	-		-	-	-	-	-	-
*trans*-Coutaric acid	0.001	0.001	0.001	0.001	0.001	-	-	-		-	-	-	-	-	-
*trans*-Resveratrol	-	-	-	-	-	0.255	0.028	0.164		0.013	0.105	0.109	0.007	-	-
Vanillic acid	-	-	-	-	-	-	-	-		-	-	-	-	0.073	0.075

#### 3.1.2. Climate Conditions

Terroir was defined by Seguin in 1988 [71] as an interactive ecosystem which includes climate, soil and the vine (rootstock and cultivar). Some authors [72] have included human factors as well, like viticultural and ecological practices. As observed in Table 1, the phenolic composition of Cabernet Sauvignon varies depending on the different origins. Brezoiu et al. [66] chose Romania from the black sea zone as the location for their research. The climate of this zone could be characterized as continental, which means mild winters with average temperatures of −1 °C and frequent snowfall. On the other hand, summer is quite warm with temperatures of around 26 °C, and this is the rainiest season of the year, with storms that can cause floods. González de Peredo et al. [67] selected different varieties with multiple countries of origin but all grown in Alcalá de Henares (Spain). In this zone, the summers are very hot, dry, and cloudless and the winters are long, cold, and partly cloudy. The temperatures can vary from 1 °C to 33 °C. Iacopini et al. [68] performed their research in Arezzo (Italy), which is characterized by hot and cloudless summers and long, cold and cloudy winters. The temperatures vary from 0 °C to 31 °C. Lastly, Obreque-Slier et al. [69] evaluated the phenolic content of the Cabernet Sauvignon variety from Maule Valley (Chile). This region presents a Mediterranean climate with temperatures between 7 °C in winter and 30 °C in the summer months. Snowfall is not usual in this zone but rain is, with an average amount of 735 mm per year.

The results observed in Table 1 exhibited a higher concentration of phenolic compounds in varieties grown in a climate of long winters with low temperatures and possible snowfall. In this case, the average phenolic compounds obtained from the Cabernet Sauvignon variety was 1.35 mg/g grape from those grown in Italy, while respective concentrations of 0.82, 0.17, and 0.11 were found in the grapes from Turkey, Chile and Spain. As an example, the concentration of catechin in the Cabernet Sauvignon grapes was 0.184 mg/g grape in those from Turkey, 0.026 mg/g in those from Spain, 1.418 mg/g in those from Italy, and 0.051 in those from Chile. However, a total comparison could not be performed as only one variety was analyzed from each different origin.

Different researchers have evaluated the effect of some climate parameters on the phenolic concentration in grapes. Van Leeuwen et al. [73] evaluated the influence of climate conditions, soil and cultivar on grape composition. To this end, three *Vitis vinifera* varieties were selected (Merlot, Cabernet Sauvignon, and Cabernet Franc) and cultivated in vineyards located in the Bordeaux region with three kinds of soils (gravelly soil, heavy clay, and sandy soil) and over three years. The climate conditions of the three years were measured by a weather station located in the same vineyard. The climate exhibited the most influential effect on the grape composition followed by soil and cultivar. The results proved that vine water availability is a decisive parameter for the phenolic compound concentration, even more than temperature and sunshine hours. In fact, a negative water balance from flowering to harvest combined with soils with water deficits resulted in earlier shoot-growth slackening, a reduced berry size and thus a high phenolic compound concentration, which increased grape quality. These results were in accordance with the observed by Ratiu et al. [74] who evaluated the influence of different climatic conditions on secondary metabolites such as phenolic compounds. They proved that conditions with low temperatures and deficiency of water facilitate the production of the phenolic compounds and especially the anthocyanin accumulation. These authors also observed that soils with nutrient stress or rich in sucrose (which induces osmotic stress) have an important influence on the synthesis of phenolic compounds.

De Oliveira et al. [70] evaluated the influence of climate conditions on the composition of Syrah grapes (Table 1). To this end, the Syrah variety grown in two zones from Brazil was selected. The first zone, Pernambuco, is characterized by latitudes 8 and 9° parallels at 350 m asl (above sea level) and with soils classified as red-yellow argisols and a semi-arid tropical climate. The second zone was Morro du Chapéu in the Chapada Diamantina region at the 11° parallel, at 1.100 m asl. The soils were classified as red-yellow deep latosols with a pH around 4.8, and the climate is classified as tropical with rain distributed throughout the year. The Syrah grapes cultivated in the Diamantina region showed a higher concentration of most of the phenolic compounds, evidence that, in the case of Brazil, a tropical climate and latosols present in soils promote a higher production of these compounds.

Fernández-Marín et al. [75] evaluated the effect of variety and terroir on stilbene concentration. The authors analyzed four different red grape varieties (Syrah, Merlot, Cabernet Sauvignon, and Pinot Noir) from four different Andalusian zones (Jerez, Cabra, Cadiar, and Ronda). The results showed that the terroir effect was stronger than the variety on stilbene concentration and that the Cabra terroir produced the highest stilbene content.

Ramos and Martínez de Toda [76] studied the influence of climate conditions and soil composition on the phenolic content in Tempranillo grapes. The study was carried out in La Rioja (Spain) in three different zones: two in La Rioja Alta with an Atlantic influence, and one in La Rioja Oriental with a Mediterranean influence. The three zones exhibited different soil compositions (clay, sand, silt and coarse elements were selected for the evaluation). In addition, the trial was carried out from 2008 to 2018, ensuring significant differences in climate conditions. The results showed a higher concentration of phenolic compounds in climate conditions with high temperatures and an important water deficit, in agreement with Van Leeuwen et al. [73,77].

In agreement with these results, many authors [78,79,80] have confirmed that high temperatures and a water deficit as a consequence of permeable soils which enhance this situation result in a lower development of the vine, leading to a higher concentration of phenolic compounds in the grapes. In addition, Del-Castillo-Alonso et al. [81] have recently proved that a supplement of ultraviolet radiation via a mechanical method increases the phenolic concentration of grape skins and consequently of the wine produced.

Furthermore, there are climate events that could directly affect the composition of grapes such as heatwaves, characterized by an important increase in temperatures with a low humidity grade. These are unpredictable episodes that can last from a day to several weeks. The drastic change of temperatures and the high values reached have an important impact on agriculture, including viticulture, with decisive desiccation of the plants and fruits and with significant changes in their composition, which could generate irreversible damage. For this reason, it is very important to evaluate the influence of heatwaves on grape compositions to predict their effect on the wine. Gouot et al. [82] evaluated the influence of heatwaves on the phenolic compositions of grapes. The authors exposed grapes to three different temperatures (ambient, high, and very high) under experimental conditions for varied periods of time (from 3 to 39 h). In the case of the high temperature, an increase in anthocyanins, flavanols and tannins was detected after just 18 h of exposure. Anthocyanins were the most affected polyphenol compounds, increasing from 0.2 mg/g to 17.1 mg/g in just 18 h. Tannins were measured in the seeds too, but no significant difference was observed at different temperatures or time of exposure, likely due to the protection conceded by the pulp and skin. It was concluded that the maximum temperature reached was a more influential factor than the exposure time. In fact, temperatures around 43 °C to 46 °C led to an increase in compounds such as anthocyanins, flavonols or tannins in just 18 h. However, temperatures higher than 53 °C resulted in a decisive degradation of compounds without any significant influence of the time of exposure. The authors suggested that in the event that a vineyard suffered a heatwave with temperatures higher than 53 °C, an early harvest would be a good decision. Nevertheless, this research should be performed in a vineyard to establish a definitive conclusion.

#### 3.1.3. Organic and Conventional Cultivation

During the last few years, the concern of consumers by the chemicals present on the food products has increased notoriously. For this reason, there are multiple vineyards that support organic viticulture practices. Organic agriculture is characterized by not using pesticides during the cultivation [83], which suppose that vineyards suffer more fungal infections and thereby producing higher levels of secondary metabolites for their defense. 

Dani et al. [84] evaluated the influence of organic and traditional cultivate on varieties Bordo and Niagara from *Vitis Labrusca*. Phenolic compounds were quantified on grape juices. The results proved that organic juices exhibited a higher total polyphenol content compared to juices from conventional grapes. It is for example the case of Bordo variety that exhibited concentrations of 290.26 and 109.71 mg/L of the measured anthocyanins in organic and conventional grape juices, respectively. In the case of catechins, the conventional grape exhibited concentrations of 2.06 mg/L and 2.13 mg/L for catechin and epicatechin whereas the organic grapes showed contents of 33.89 and 2.72 mg/L for catechin and epicatechin. For the procyanidins (B1-B4) the conventional cultivation showed a concentration of 15.77 mg/L and the organic one a concentration of 20.52 mg/L. In the case of Niagara variety, the conventional cultivation displayed 7.39 and 5.95 mg/L for catechin and epicatechin whereas the organic cultivation showed concentrations of 0.90 and 1.81 mg/L, respectively. Conventional cultivation showed concentrations of procyanidins (B1-B4) of 24.35 mg/L and 27.12 mg/L for organic cultivation. 

Angelica De Pascali et al. [85] evaluated the use of conventional and organic practices as well on Apulia Negroamaro variety. The results were in consonance with the previously observed, a significant increment of phenolic compounds was detected when organic cultivation was used. J.Olejar [86] suggested the use of weedmat undervine treatment instead of herbicides treatment on the vineyard and evaluated its influence on phenolic composition grapes. The grapes from conventional cultivation exposed a total phenolic content of 1.82 mg/L whereas the organic cultivation showed contents of 1.98 and 2.03 mg/L for black weedmat and white weedmat, respectively.

Other authors [87,88,89,90,91] have evaluated the influence of organic practices on grape composition and the results have proved a promotion on the production of phenolic compounds but, it was observed as well that its influence is highly related to the variety.

#### 3.1.4. Biostimulants

Plant biostimulants are defined at European level as “products stimulating plant nutrition processes independently of the product’s nutrient content, with the aim of improving the crop quality traits among other characteristics of the plant” [92]. Biostimulants have been widely used in vineyards with the aim of promoting the production of some compounds in grape such as phenolic compounds. Martínez-Gil et al. and Pardo-García et al. used different concentrations of an aqueous oak extract on Verdejo, Petit Verdot, and Monastrell grapes varieties [93,94,95]. The extract was diluted with water to one in four parts and applied (i) once, on the seventh day post-veraison (25%-1 treatment) and (ii) four times (7, 11, 15, and 18 days post-veraison (25%—four treatments). Lastly, the undiluted extract was tested once on the seventh day post-veraison (100%—one treatment). A total of 16 plants were selected in the same row, with other rows with untreated plants between the different applications. A total of 300 mL of the corresponding formulation were sprayed onto the leaves of each plant. The treatments were carried out at 20 °C and the winemaking process was performed under traditional conditions. The results showed that the phenolic compounds were correctly assimilated by the grapes and transmitted into their wines with positive effects on their final quality. Table 2 shows the phenolic compound concentrations detected in the Monastrell wine after the oak extract treatment. The treatment significantly increased the total content of hydroxycinnamic acids and hydroxycinnamoyltartaric acids in the wines. These compounds were more abundant in the treated wines than in the control, especially after malolactic fermentation. On the other hand, after 6 months of malolactic fermentation, a decrease in trans-caftaric and trans-coutaric acids was observed and an increase in trans-caffeic and p-coumaric acids, with the greatest content found in the grapes following the 25%—1 and 25%—4 treatments. These results support the idea that wine from treatments with more oak extract are more resistant to oxidation. In addition, total hydroxybenzoic acids increased as well with aging, with gallic acid being the most abundant compound. Regarding stilbenes, the oak treatment led to an increase in the content of trans-resveratrol and trans-piceid in the wine. In the case of the anthocyanins, the acylated anthocyanins content was significantly higher when 100%—1 treatment was applied compared with the control, but they were found to decrease in all the wines over time. No effect of the oak extract treatment on the concentration of vitisins A and B was detected. However, significant differences in the main flavanol concentrations ((+)-catechin and (−)-epicatechin) were perceived among the treated wines and the control. The 100%—1 treated wines exhibited the highest content of total phenolic compounds and the wines from the 25%—1 treatment the lowest. Furthermore, a relationship was found between the oak extract treatment and the increase in flavanols in general.

Sánchez-Gómez et al. [96,97] used aqueous extracts from Moscatel grapevine shoots as a biostimulant on Airén white grapevines. A positive influence of the grapevine shoot extract was detected, with a significant increase in volatile and phenolic compounds, especially phenolic acids. The resulting wines exhibited a characteristic fruity and floral aroma and there was a significant contribution of toasty and spicy notes. Cebrián et al. [98] added two *Vitis vinifera* vine-shoots (Airén and Cencibel) in two formats (chip and granule) at a concentration of 12 g/L during winemaking. The results (Table 2) show that the content of phenolic compounds increased when vine-shoots were used. In the case of the Airén wine, the phenolic content was higher when granules were used instead of chips due to the higher contact surface, especially before alcoholic fermentation. In the case of the Cencibel wine, when vine-shoots were added before alcoholic fermentation, an increase in its phenolic concentration was observed when chips were used, whereas a decrease in content was detected when granules were employed. On the other hand, when vine-shoots were added after alcoholic fermentation, no significant differences were observed with chips, but a significant increase was observed when granules were used.

Other biostimulants have also been used to improve the phenolic composition of wines. Pardo-García et al. [99,100] studied the use of eugenol, guaiacol and the combination of both in foliar applications on Monastrell grapevines. All the treatments increased the concentration of phenolic compounds in the wines, but the combination of both compounds (eugenol + guaiacol) significantly increased the concentrations of anthocyanins and stilbenes. Brillante et al. and Dinis et al. [101,102] suggested the use of kaolin (a particle film technology) as a biostimulant in the vineyard, the results presenting an increase in the phenolic compounds content and the resulting wines being considered more attractive and appreciated by the tasters. The total flavonoids quantified were 2.205 mg/L for the control sample and 2.830 mg/L for the vineyards treated with kaolin. Furthermore, the anthocyanins quantified were 0.75 mg/L in the control samples and 0.97 mg/L for the treated samples. In addition, Brillante suggested the use of pinolene (a film-forming antitranspirant) in the vineyard as a biostimulant, but a decrease in phenolic compounds, especially anthocyanins, was observed, and the final wine was less appreciated by the tasters. Lastly, methyl jasmonate, chitosan and yeast extract have been evaluated as biostimulant treatments in vineyards. Portu et al. [65,103] evaluated the use of the three treatments on Tempranillo and Graciano varieties. The results are shown in Table 2. Methyl jasmonate and yeast extract had a significant impact on the concentration of phenolic compounds, especially anthocyanins and stilbenes. However, the chitosan treatment did not have a significant effect on phenolic compounds. In contrast, other authors have attributed to chitosan important biostimulant properties [104]. Portu et al. [105] also researched the treatment of Garnacha grapevines with methyl jasmonate combined with phenylalanine. However, the phenolic content was not improved after the treatment in comparison with the control sample (Table 2). The result was similar when the treatment was repeated with only phenylalanine. Nevertheless, Ali Andi et al. [106] also performed research based on the use of methyl jasmonate and phenylalanine in *Vitis vinifera* L. cv. Shahani variety under different external light conditions. The results showed that the combination of both compounds under darkness growth conditions produced the highest production of total phenolic compounds, flavonoids and stilbenes, even higher than when methyl jasmonate was applied under light conditions. Other authors have suggested the use of benzothiadiazole as a biostimulant with good results in the production of phenolic compounds in grapes [107,108,109]. It has been tested in multiple varieties such as Monastrell, Merlot and Cabernet Sauvignon, with similar results to those obtained with methyl jasmonate, these two biostimulants being the most extensively recommended by many authors. Fernandez-Marin et al. [110] also evaluated the use of three different preharvest treatments: benzothiadiazole, methyl jasmonate, and chitosan on stilbene concentrations in the Syrah variety. After harvesting, these treatments were combined with UVC treatment. The results showed that benzothiadiazole significantly increased the *trans*-resveratrol concentration in grapes, but this appears to be linked to a ripening delay. When these treatments were combined, only the methyl jasmonate combined with UVC treatment was successful in increasing the stilbene content of the grapes.

In conclusion, it is important to remark that many of these results are influenced by the variety of study and by the climatic conditions, very influential variables in the foliar treatment with biostimulants. Biostimulants obtained from plants, such as vine-shoots or oak extracts, have proved to be a useful tool for increasing the concentration of phenolic compounds in grapes and wines. Other biostimulants such as eugenol, guaiacol, methyl jasmonate, chitosan, or benzothiadiazole have been evaluated as biostimulants in winemaking, all of them demonstrating an increase in phenolic compound concentrations.

**Table 2:** Pardo-García et al. and Cebrián et al.; Concentration of phenolic compounds in wines (mg/L) from Monastrell, Airén and Cencibel varieties after grapevine/wine treatment with oak extracts. * 100%: one treatment with oak extract on seventh day post-veraison; ** BAF: Before alcoholic fermentation, AAF: after alcoholic fermentation, AMF: after malolactic fermentation; Portu et al.: Concentration of phenolic compounds in wines (mg/L)/grapes (mg/kg) from Garnacha and Tempranillo varieties after grapevine treatment with phenylalanine (Phe), methyl jasmonate (MeJ), chitosan (Ch), and yeast extracts (YE); n.d.: not detected; -: this compound has not been analyzed during this research.

**Variety**	**Monastrell**		**Airén**						**Cencibel**					
Reference	Pardo-García et al. [93]		Cebrián et al. [98]											
Compound(mg/L)/Treatment	Oak extract		Control BAF **	Chips BAF **	Granule BAF **	Control AAF **	Chips AAF **	Granule AAF **	Control BAF **	Chips BAF **	Granule BAF **	Control AMF **	Chips AMF **	Granule AMF **
	Control	100% *												
*trans*-Caffeic acid	0.74	1.68	2.815	2.51	2.69	3.49	3.93	3.48	0.36	0.44	0.33	0.35	0.50	0.78
*trans*-Caftaric acid	17.9	25.1	7.61	6.12	8.31	6.18	4.85	5.68	43.85	46.12	45.84	43.55	44.24	45.25
(+)-Catechin	29.3	49.3	9.50	9.59	12.21	7.25	9.99	11.50	22.13	25.37	20.55	18.05	18.83	21.70
*p*-Coumaric acid	0.68	2.79	-	-	-	-	-	-	-	-	-	-	-	-
*trans*-Coutaric acid	5.34	10.6	3.87	3.76	3.68	3.65	3.06	2.93	23.71	24.70	24.85	21.57	21.22	20.69
Cyanidin 3-(6-*p*-coumaroyl)-glucoside	1.33	0.85	-	-	-	-	-	-	-	-	-	-	-	-
Cyanidin 3-*O*-glucoside	0.87	0.82	-	-	-	-	-	-	-	-	-	-	-	-
Delphinidin 3-*O*-glucoside	11.2	11.3	-	-	-	-	-	-	-	-	-	-	-	-
(−)-Epicatechin	12.4	31.2	15.21	16.09	32.41	13.03	15.62	35.56	132.16	155.51	129.25	65.40	64.64	83.37
Gallic acid	22.3	45.8	5.65	7.24	8.18	6.55	7.18	8.49	16.42	17.52	17.93	18.84	16.08	19.15
Malvidin 3-*O*-glucoside	148	111	-	-	-	-	-	-	-	-	-	-	-	-
Malvidin 3-(6-*p*-coumaroyl)-glucoside	17.5	15.1	-	-	-	-	-	-	-	-	-	-	-	-
Malvidin 3-*O*-(6-acetyl)-glucoside	12.2	10.8	-	-	-	-	-	-	-	-	-	-	-	-
Myricetin 3-*O*-galactoside	4.6	3.9	-	-	-	-	-	-	-	-	-	-	-	-
Myricetin 3-*O*-glucuronide + myricetin 3-*O*-glucoside	24.5	10.4	-	-	-	-	-	-	-	-	-	-	-	-
Peonidin 3-*O*-(6-acetyl)-glucoside	1.02	0.79	-	-	-	-	-	-	-	-	-	-	-	-
Peonidin 3-(6-*p*-coumaroyl)-glucoside	3.76	2.88	-	-	-	-	-	-	-	-	-	-	-	-
Peonidin 3-*O*-glucoside	6.2	4.4	-	-	-	-	-	-	-	-	-	-	-	-
Petunidin 3-*O*-glucoside	26.8	21.4	-	-	-	-	-	-	-	-	-	-	-	-
Procyanidin B2	-	-	n.d.	n.d.	n.d.	3.67	3.14	3.98	1.96	1.22	1.41	1.78	0.74	1.38
Quercetin	14.5	23	1.24	1.12	1.29	2.06	1.08	n.d.	1.87	1.43	1.15	1.17	1.27	1.07
Quercetin 3-*O*-glucoside	4.69	3.38	-	-	-	-	-	-	-	-	-	-	-	-
Quercetin 3-*O*-glucoronide	-	-	2.76	2.33	2.12	2.52	1.95	2.41	4.03	4.08	3.64	4.19	3.75	2.60
*trans*-Resveratrol	0.15	0.4	0.36	0.84	3.93	0.36	1.00	3.94	0.78	0.88	1.62	0.64	0.87	1.86
Syringetin 3-*O*-glucoside	3.94	3.65	-	-	-	-	-	-	-	-	-	-	-	-
Syringic acid	7.2	7.9	-	-	-	-	-	-	-	-	-	-	-	-
Vanillic acid	2.98	2.04	n.d.	0.19	0.27	n.d.	n.d.	n.d.	1.67	1.67	1.76	2.06	1.67	1.90
**Variety**			**Garnacha**		**Tempranillo**									
Reference			Portu et al. [103,105]											
Compound (mg/L)/treatment			Control	Phe	MeJ	Phe + MeJ	Control	MeJ	CHT	YE	Control	MeJ	CHT	YE
							Grape berries (mg/kg)				Wines (mg/L)			
*trans*-Caffeic acid			-	-	-	-	n.d.	n.d.	n.d.	n.d.	4.48	4.75	4.61	4.59
*trans*-Caftaric acid			87.19	98.7	116.08	100.53	28.53	33.72	30.73	26.07	43.43	38.46	41.34	35.18
(+)-Catechin			123.13	105.51	108.45	112.49	27.48	29.94	24.65	24.76	12.35	12.13	11.57	10.38
*p*-Coumaric acid			-	-	-	-	n.d.	n.d.	n.d.	n.d.	1.48	1.54	1.37	1.22
*trans*-Coutaric acid			18.82	24.91	29.41	24.55	35.55	37.35	35.54	33.34	32.69	29.51	32.75	25.16
Cyanidin 3-(6-*p*-coumaroyl)-glucoside			2.72	2.77	3.34	2.91	10.4	13.25	10.31	11.96	1.84	2.36	1.94	1.83
Cyanidin 3-*O*-glucoside			6.58	5.73	15.12	8.04	34.16	56.4	37.22	50.1	1.84	2.7	1.96	1.81
Delphinidin 3-*O*-glucoside			24.74	29.66	58.12	36.58	261.6	319.98	264.77	312.3	36.17	48.79	39.59	38.5
(−)-Epicatechin			59.02	44.72	37.92	45.31	17.36	18.48	16.08	18.28	5.9	6.93	5.14	5.39
Gallic acid			14.65	18.89	19.3	16.23	9.21	9.98	8.5	8.35	11.71	11.11	11.82	10.82
Malvidin 3-*O*-glucoside			384.58	430.53	525.75	474.32	541.57	577.92	535.57	618.94	280.43	310.57	286.58	315.54
Malvidin 3-(6-*p*-coumaroyl)-glucoside			3.1	3.03	3.03	3.15	73.24	96.75	68.01	95.24	9.03	10.06	9.65	9.39
Malvidin 3-*O*-(6-acetyl)-glucoside			11.91	13.34	14.1	13.08	35.36	33.68	33.46	36.89	16.88	17.07	16.56	18.43
Myricetin 3-*O*-galactoside			0.42	0.45	0.74	0.57	7.4	7.12	6.3	7.28	1.54	1.57	1.28	1.42
Myricetin 3-*O*-glucuronide + myricetin 3-*O*-glucoside			5.18	5.84	6.82	6.03	7.98	8.44	6.76	8.24	2.12	1.85	1.57	1.68
Peonidin 3-*O*-(6-acetyl)-glucoside			11.36	11.29	14.03	12.04	3.29	3.87	3.44	3.92	1.05	1.23	1.08	1.07
Peonidin 3-(6-*p*-coumaroyl)-glucoside			3.02	2.9	3.28	3.01	23.17	25.63	22.33	24.95	5.94	7.39	6.63	6.29
Peonidin 3-*O*-glucoside			51.97	43.02	88.67	60.19	71.25	101.24	72.86	93.25	8.93	14.37	10.16	9.5
Petunidin 3-*O*-glucoside			30.3	35.68	60.97	43.01	190.18	225.35	190.02	222.11	55.47	69.72	59.3	62.21
Procyanidin B2			27.33	22.26	20.37	21.13	5.19	6.61	5.09	5.5	3.63	4.34	3.63	3.05
Quercetin 3-*O*-glucoside			17.23	16.75	30.61	28.29	23.71	30.99	20.11	29.72	n.d.	n.d.	n.d.	n.d.
Quercetin 3-*O*-glucoronide			9.53	16.01	22.54	22.49	17.42	21.29	14.86	21.1	4.95	4.34	3.29	3.85
*trans*-Resveratrol			3.53	3.26	6.8	6.55	0.13	0.37	0.32	0.39	0.32	0.27	0.32	0.25
Syringetin 3-*O*-glucoside			0.53	0.49	0.69	0.71	4.81	4.89	4.32	4.69	2.16	2.16	1.89	2.1
Syringic acid			3.93	2.65	4.2	4.47	-	-	-	-	-	-	-	-
Vanillic acid			3.79	2.85	3.3	4.27	-	-	-	-	-	-	-	-

### 3.2. Wine Elaboration

There are a variety of factors that could influence the phenolic compound content in grapes. During winemaking, it is possible to distinguish the internal and external agents that have an impact on phenolic compound development. Three stages have been defined during the winemaking process: (i) pre-fermentative, (ii) fermentative, and (iii) post-fermentative. Pre-fermentative maceration and thermovinification are strategies that have been selected for study in the pre-fermentative stage; yeast strain and additives were the variables studied during the fermentative process; and lastly, fining agents, filtration and post-fermentative maceration have been studied for the post-fermentative stage.

#### 3.2.1. Pre-Fermentative Maceration

*Pre-fermentative maceration* is the period during red and rose winemaking in which the solid parts of the grapes are in contact with the must before alcoholic fermentation takes place. This process is of vital importance because interesting compounds are transferred from the grape skins into the must, increasing their concentration in the wine. First, the grapes are crushed ensuring the breakdown of the vacuole and cell membranes [111]. Then, the enzymatic activity of the pectinase, protease and polysaccharides added during the maceration process ensure the cell wall degradation [112]. Finally, the contact of solid parts with the must promotes the extraction of compounds, including phenolic compounds. 

Multiple factors affect the maceration process, the temperature being one of the most important. Maceration has to be carried out under low temperatures to prevent yeast growth and thus the start of the fermentation process since some phenolic compounds are better extracted in the absence of ethanol. This process is known as cold maceration and some authors have recommended that it takes place at temperatures around 4–8 °C [113]. Many authors have reported a significant increase in phenolic compounds in the must when cold maceration was performed in a vast range of grape cultivars [114,115,116,117,118]. Gómez-Miguez et al. [119] noticed a higher extraction of anthocyanins than other phenolic compounds, associated with the high solubility of anthocyanins in aqueous medium, the better protection of the anthocyanins at lower temperatures and lower levels of tannins [120]. Hydroxycinnamic acids and flavonols exhibited a similar growth rate to anthocyanins but flavonols showed a slower process, associated with their lower solubility in aqueous medium. Furthermore, an increase in catechin, epicatechin and tannin concentrations was also observed when the maceration process was extended. Nevertheless, after a certain time the extraction rates slowed down indicating a possible saturation of the aqueous medium [121]. Lastly, a decrease was observed in the concentration of phenolic compounds a certain time after the maceration finished, related to the stabilization of the increased compounds. For this reason, the use of additives or enzymes treatments that could ensure a successful maceration process has been recommended [122,123,124,125].

As previously mentioned, grape skins, especially in red varieties, are rich in phenolic compounds such as anthocyanins and tannins. However, some authors have shown that less than 50% of these compounds are transferred to wine during winemaking [126]. This phenomenon is closely related to the limited permeability of cell walls and cytoplasmatic membranes [127,128]. For this reason, numerous pre-fermentative techniques have focused on weakening the cell barriers and increasing the polyphenol content in wines in recent years. 

*Carbonic maceration* is the process whereby the grapes undergo maceration under anaerobic conditions [129,130]. Grapes (without destemming or crushing) are placed in tanks and subjected to a carbon dioxide atmosphere. In these conditions, intracellular fermentation takes place inside the grapes and starts the production of alcohol and the degradation of malic acid. In addition, volatile compounds are formed and phenolic compounds are diffused from the skin to the pulp [131]. At a certain moment, the grape skin is broken and its juice is transferred and mixed with the other juices, which are fermented by yeasts. It is important to remark that the internal fermentation of the grapes and the alcoholic fermentation of the must occur simultaneously. After the alcoholic fermentation, must is removed and the grapes are pressed. The two musts undergo alcoholic fermentation mixed together or separately. Finally, they are subjected to malolactic fermentation. González-Arenzana et al. [132] analysed a total of 84 wines from D.O.Ca. Rioja of the Tempranillo *Vitis vinifera* variety of the same vintage (2017). Forty of these wines had been elaborated by carbonic maceration and forty four were elaborated by the traditional methodology. No significant differences were appreciated in their physical-chemical composition or microbial load. However, the wines made by carbonic fermentation presented a higher content of phenolic compounds. Even if the anthocyanin concentration of the carbonic maceration wines was similar to those of the wines elaborated by traditional methodologies, the concentrations of vitisins A and B were considerably increased by carbonic maceration. The authors recommended the use this alternative maceration to the traditional methodology in order to increase the phenolic compound concentration in wine.

It is common for enzymes to be added during pre-fermentative maceration. Pectolytic enzymes are exogenous preparations of pectinases, hemicellulases and cellulases added during the fermentation process that attack the grape cell walls allowing the release of intracellular pigments [133]. For this reason, an increase in the phenolic compound concentration was expected. In fact, some authors have shown a significant rise in the anthocyanin content when pectolytic enzymes were added [134,135]. However, other authors have found the opposite effect [136], a fact highly related to the enzymatic preparation and maturity of the grape.

#### 3.2.2. Thermovinification and Pulsed Electric Field

*Thermovinification* is a pre-fermentative methodology that makes it possible to disrupt the cell structure when temperatures higher than 70 °C are used for a short period of time (30–40 min) and followed by a cooling process before the alcoholic fermentation. This process involves water soluble phenolic compounds coming out from the cells [137]. El Darra et al. [134] performed a research study where Cabernet Sauvignon grapes underwent a thermovinification process at 70 °C for 30 min before cooling until 20 °C. The musts were analyzed by HPLC-DAD, and the phenolic compounds, mainly anthocyanins and flavonols, were quantified. The results showed that the content of anthocyanins was not statistically different after the thermovinification process but that the content of total phenolic compounds and total flavonols were statistically higher when thermovinification was applied. Geffroy et al. [138] studied the influence of temperature and heating time on the phenolic content of *Vitis Vinifera* L. Carignan. To this end, grapes were exposed to temperature levels of 50 °C and 75 °C and two heating times (30 min and 180 min). The results showed that the heating temperature had a significant impact on the extraction of phenolic compounds, and a thermal degradation of anthocyanins was detected when the temperatures exceeded the 75 °C. It was also observed that the reduction in the heating temperature could be compensated for by extending the heating time. Thus, the musts obtained from grapes heated at 50 °C for 180 min had a similar level of phenolic compounds as those treated at 75 °C for 30 min.

*Pulsed electric field* is a non-thermal treatment inducing pores in cell membranes, thus achieving the breakdown potential, increasing their permeability and enhance the exchange of intercellular compounds [139,140]. Samples are exposed to a high-intensity electric field (5–10 kV/cm) for short durations (30 min). This technology has been tested on multiple *Vitis Vinifera* varieties such as Cabernet Sauvignon, which was selected by El Darra et al. [134] to evaluate its use. The results indicated that the use of this technology at a moderate intensity during cold maceration significantly increased the concentration of phenolic compounds, especially anthocyanins, in comparison with when this treatment was used during alcoholic fermentation. An increase in the tannin concentration in wine was also found when pulsed electric field treatment was used on the second or fourth day of cold maceration. Saldaña et al. [141] also evaluated this technique for extracting polyphenols from Grenache, Syrah and Tempranillo grapes. The results showed that the treatment increased the extraction of polyphenols by more than 40% in the Syrah and Grenache varieties but lowered it (24%) in Tempranillo grapes. The greatest effect was observed at the highest electric field strength and applying longer pulses. Other authors [134,142,143,144] have evaluated the use of the pulsed electric field methodology, in every case observing a rise in the phenolic compound content of the must and wine.

Wojdyło et al. [126] performed a comparative research study of different pre-fermentative maceration processes. The authors selected the *Vitis vinifera* Dornfelder variety cultivated in Poland. Four different treatments were examined. One of them was the traditional process used as a control. For the other three, samples were macerated in a first trial. After that, one of them was subjected to microwave extraction for eight minutes at 1200 W and 80 °C. Another was heated at the same temperature, but any extra treatment was carried out corresponding to the thermomaceration. Finally, in the last trial, a pectinolytic enzyme was added at a dose of 0.05 mg/L for 1 h at 50 °C. After this time, the must was heated until 65 °C for 2 min to denature the enzyme. All the samples after pre-treatment were cooled in an ice bath prior to adding yeast and starting the fermentation process. The authors detected a significant influence of the pre-treatment on the phenolic compound composition in the must/wine. In this case, the samples pre-treated with microwaves exhibited the highest phenolic compound concentration (43.44 mg/mL), followed by the enzyme treatment (34.07 mg/mL). No significant differences were found in the musts (before starting fermentation) of all the pre-treatment maceration samples and the control, but the content of phenolic compounds was slightly higher in the must after thermomaceration (29.79 mg/mL) than in the control sample (28.95 mg/mL). 

#### 3.2.3. Yeast Strain and Bacteria

Phenolic compound content is usually modified through enzymatic reactions or the metabolic activities of yeast [145]. For example, *β*-glucosidase is responsible for the hydrolysis of glycosidic linkages. Furthermore, yeast may react with anthocyanins to form pyranoanthocyanins such as vitisins [146], but a high hydroxycinnamate decarboxylase activity is required for effective production [147]. Lastly, it has been reported that anthocyanins are usually adsorbed on the yeast cell wall, which could be of great importance in grape varieties with a lower anthocyanin content [148].

Although *Saccharomyces cerevisiae* yeast is commonly used with good results [149], during the last few years other *Saccharomyces* and non-*Saccharomyces* species have been evaluated [150]. To this end, non-*Saccharomyces* strains, that exhibited a positive hydroxycinnamate decarboxylase activity, such as *Torulaspora delbrekii*, *Pichia guilliermondii* or *Schizosaccharomyces pombe* have been used to start alcoholic fermentation [146,151,152].

Topić Božič et al. [153] evaluated the use of 95 different *Saccharomyces* and non-*Saccharomyces* strains on the alcoholic fermentation of the Pinot Noir variety. The hydroxycinnamate decarboxylase activity of the selected strains varied from 0.0% to 91.1%, ensuring the heterogeneity of the group. All the non-*Saccharomyces* strains except *P. manshurica* M49 produced higher concentrations of vinylphenolic pyranoanthocyanins than the *Saccharomyces* strains. In fact, the *Pichia guilliermondii* ZIM624 and *Wickerhamomyces anomalus* S138 strains showed the highest production of vinylphenolic pyranoanthocyanins, with 40.2 and 38.5 mg/L, respectively. In addition, the authors observed for the first time pyranoanthocyanin formation by the *S. paradoxus* strain. 

Minnaar et al. [154] evaluated the use of mixed cultures of yeast + malolactic bacteria such as *Saccharomyces* + *O. oeni/Lb. plantarum* and *Saccharomyces* + non-*Saccharomyces* + *O. oeni/Lb. plantarum* in Syrah grape must during alcoholic fermentation. The authors included a fermentation by *S. cerevisiae* as a reference. The results showed that the phenolic content of wines elaborated with mixed strains was 1 or 3 times higher than the wines elaborated with *Saccharomyces*. Hence, the authors proposed the mixed culture of *S. cerevisiae* and malolactic bacteria to improve the phenolic content of Syrah wines. In addition, no negative effects of mixed cultures on the vinification process were detected. Many authors have evaluated the influence of yeast strain on the phenolic composition and organoleptic properties of wines after alcoholic fermentation. All of them have concluded that a clear correlation exists, a specific study for each variety being required. In addition, the results suggest the possibility of using non-*Saccharomyces* or mixed cultures as feasible strategies for increasing the phenolic composition of final wines.

#### 3.2.4. Additives

Additives are chemical or natural substances that are added during the winemaking process with multiple functions. It is for example the case of sulphur dioxide (SO_2_), which has antioxidant and antimicrobial properties and is the most used preservative in the wine industry. It has proved to inhibit polyphenol oxidase activity during winemaking [155], preventing the oxidation of phenolics and, therefore, the loss of the polyphenols in the wine. However, several human health risks have been associated with the consumption of SO_2_, including dermatitis, urticaria, angioedema, diarrhea, abdominal pain, bronchoconstriction, or anaphylaxis [156]. In fact, the maximum SO_2_ concentration in wine authorized by the International Organization of Vine and Wine (IOV) was 200 mg/L for white and rosé wines, and 150 mg/L for red wines [157]. 

The IOV described in the resolution IOV-OENO 439 in 2012 [157] the additives with different purposes it allows to be used in wines. In the case of sweeteners, dextrose, fructose, invert sugar and other derivates of sugar are allowed with the aim of balancing the gustatory properties of a wine. To increase the alcoholic content or dilute another additive, ethyl alcohol or wine distillate are recommended. In addition, additives such as lactic acid, malic acid, tartaric acid, and citric acid are the only additives allowed by the IOV to reduce the pH of wine and to modify its gustatory properties. On the other hand, when the acidity of the wine should to be increased to achieve the quality properties required for this product, the IOV suggests the use of neutral potassium tartrate, potassium acid carbonate or calcium carbonate.

The caramel or other food coloring have been accepted to increase the yellow/red tone of wines. Lastly, when dissolving coloring or sweetener substances, adjusting the content of a final product or reducing the number of salts is required, the OIV recommend the use of water (under OMS characteristics).

#### 3.2.5. Fining Agents

Fining agents are chemical or natural compounds with a positive or negative charge that bond with proteins, tannins, and phenolic compounds which are in suspension in wine causing a cloud aspect [158]. For this reason, fining agents are commonly used to clarify and enhance wine stability. Moreover, the modulation of tannins and phenolic composition can eliminate intense flavors and soften sensory properties such as bitterness or astringency [159,160]. 

Chemical fining agents have been extensively used by multiple authors, for example polyvinylpolypyrrolidone (PVPP), which is commonly used as it has polyphenol binding affinities. Gil et al. [159] evaluated the use of this compound as a fining agent in two blends of rosé varieties: Grenache Noir and Merlot (70/30 and 50/50). Four concentrations of PVPP were evaluated, from 20 g/hl to 80 g/hl (maximum legal dose). The results (Table 3) exhibited a significant decrease in polyphenol contents, especially flavonols, flavanols (especially dimers and trimmers), and anthocyanins (especially anthocyanin coumaroyl derivatives), which is associated with a strong preference to be adsorbed by PVPP.

Most of the traditional fining agents are based on animal proteins such as casein, egg albumin, gelatin, or isinglass. However, a strong fining agent action could result in the extreme decrease in phenolic compounds with undesirable consequences, such as color losses or unpleasant aromas [161]. Furthermore, recent research has shown that animal-origin fining agents have the potential to cause allergies or intolerance, with important repercussions for the health of consumers [162]. In fact, according to EU regulation No. 579/2012 [163], all potentially allergenic fining agents in wine at a concentration higher than 0.25 mg/L must be declared on the label.

For this reason, a great deal of research is being performed into finding alternative fining agents with a lower impact on health and a controlled phenolic modulation. Río Segade et al. [164] studied the possibility of using plant proteins as fining agents. The authors selected four young wines (Primitivo, Montepulciano, Syrah, and Nebbiolo) based on their different organoleptic properties and used fining agents that were proteins of varied origins: two from peas, two from potatoes, and one of animal gelatin. In addition, one control sample (without fining agent) was included for each variety.

The highest decrease in polyphenol content with respect to the control wine was found when gelatin was used (Table 3). It is essential to remind that is the currently most used fining agent with success, so this result was expected. On the other hand, high doses of the fining agent from potato led to a significant decrease in polyphenol content. In fact, the use of potato agents at high doses enabled the reduction of polymeric and oligomeric flavanols similar to that obtained when gelatin agent was employed. Pea agents showed a decrease of polymeric and oligomeric flavanols when they were used at high doses in Syrah and Nebbiolo wine. However, the polyphenol decrease was not significantly different to that observed in the control samples. Kang et al. [165] also investigated the effect of a potato protein on the phenolic composition of Cabernet Sauvignon wines. The authors studied the influence of the wine matrix and fining parameters such as pH, ethanol concentration, sugar concentration, temperature, and agitation. The results identified that the efficacy of potato protein as a fining agent increased at higher temperatures (20 °C) and at lower pH values and alcohol concentrations. 

The conclusion was drawn that it is possible to use alternative plant-based fining agents for reducing the polyphenol content of wine, but an in-depth study should be performed into their nature and the doses required according to the variety, the origin and even the climate conditions.

**Table 3:** Total phenolic concentration (mg/L) in wines from different varieties treated with chemical, animal and plant fining agents. Percentage of reduction has been included. PVPP: polyvinylpolypyrrolidone.

Reference	Wine	Treatments	Total Phenolic Content (mg/L)	Reduction (%)
Gil et al. [159]	Rosé A	Control	156	-
		PVPP 20 g/hL	134	14
		PVPP 40 g/hL	125	20
		PVPP 60 g/hL	120	23
		PVPP 80 g/hL	114	27
	Rosé B	Control	182	-
		PVPP 20 g/hL	149	18
		PVPP 40 g/hL	135	26
		PVPP 60 g/hL	129	29
		PVPP 80 g/hL	127	30
Río Segade et al. [164]	Primitivo	Control	4577	-
		Gelatin_Low	4237	7
		Gelatin_High	3030	34
		Pea_1_Low	4198	8
		Pea_1_High	4204	8
		Pea_2_Low	4519	1
		Pea_2_High	4489	2
		Potato_1_Low	4337	5
		Potato_1_High	4539	1
		Potato_2_Low	4554	1
		Potato_2_High	4386	4
	Montepulciano	Control	4770	-
		Gelatin_Low	4261	11
		Gelatin_High	3880	19
		Pea_1_Low	4446	7
		Pea_1_High	4320	9
		Pea_2_Low	4566	4
		Pea_2_High	4484	6
		Potato_1_Low	4222	11
		Potato_1_High	4272	10
		Potato_2_Low	4701	1
		Potato_2_High	4524	5
	Syrah	Control	4753	-
		Gelatin_Low	4578	4
		Gelatin_High	4199	12
		Pea_1_Low	4512	5
		Pea_1_High	4538	5
		Pea_2_Low	4531	5
		Pea_2_High	4276	10
		Potato_1_Low	4427	7
		Potato_1_High	4147	13
		Potato_2_Low	4620	3
		Potato_2_High	4392	8
	Nebbiolo	Control	5718	-
		Gelatin_Low	5415	5
		Gelatin_High	4874	15
		Pea_1_Low	5214	9
		Pea_1_High	5147	10
		Pea_2_Low	5576	2
		Pea_2_High	5496	4
		Potato_1_Low	5254	8
		Potato_1_High	5183	9
		Potato_2_Low	5615	2
		Potato_2_High	5596	2

#### 3.2.6. Post-Fermentative Maceration

The appropriate extraction of phenolic compounds is greatly influenced by the time of maceration and the type of medium [166]. As previously described (Section 3.2.1.), in pre-fermentative maceration a high extraction is observed of anthocyanins (located in the skin) and the low-molecular-weight flavanols (located largely in the seeds). However, high-weight flavanols (located mainly in the seed as well), flavonols (in the skins), and phenolic acids are more soluble in alcoholic solution than in aqueous media [167,168]. In addition, some phenolic compounds such as gallic acid or protocatechuic acid require of long maceration period to achieve a high extraction level [169], which is why post-fermentative maceration is commonly used by multiple authors to increase the extraction of less water-soluble compounds. Nevertheless, extended maceration can lead to sensory problems related to bitterness and astringency of the wine [168]. 

Rivero et al. [170] employed overripe seeds from Pedro Ximénez grapes to evaluate the effect of post-fermentative maceration on Syrah wines. The author selected three different treatments: (i) single post-fermentative maceration (SW), (ii) double post-fermentative maceration (double addition of seeds, DW), and (iii) traditionally made wines (without post-fermentative maceration, CW). Post-fermentative maceration was performed for 30, 60 and 150 days. The results (Table 4) showed significant differences in the phenolic compound concentrations between traditional maceration (CW) and the different treatments (SW and DW). In particular, benzoic acids increased by 2% and 5%, flavanols by 8% and 22%, and procyanidins by 8% and 10% in SW and DW, respectively.

Casassa et al. [171] researched the use of extended maceration for one and six months and the addition of extra pomace during extended maceration in Pinot Noir and Zinfandel wines. The use of extended (6 months) and double maceration decreased the amount of anthocyanin and anthocyanin-derived compounds by 53% in the Pinot Noir and 63% in the Zinfadel wines, in comparison with the control wine (Table 4). The tannins content increased when 6 months of maceration was used, 13-fold in the case of the Pinot Noir and 1.6-fold in the case of the Zinfadel. However, no significant differences were observed when 1 month of extended maceration or double pomace treatments were used. These results suggest that after a maceration of 30 days or less, no more phenolic compounds can be extracted. The authors concluded that macerating for too long or adding double pomace seem to cause a decrease in interesting compounds in wine with probable negative effects on wine color and sensory properties easily noticeable by consumers. However, these treatments were notably affected by the variety employed, suggesting that this study should be extended to different varieties before making a definitive conclusion.

Finally, Francesca et al. [172] studied the influence of post-fermentative maceration on phenolic compounds in Aglianico wine that underwent post-fermentative maceration with the grapes own skin and seeds for up to 90 days. The results (Table 4) showed that the total phenol content varied with the time of contact with the wine. The highest concentration of total anthocyanins (241.48 mg/L) was recorded 50 days of post-fermentation maceration. In general, the phenolic content increased with maceration time, reaching a maximum value (854.9 mg/L) on day 90 of the post-fermentation maceration. However, the rate of increase was higher during the first 40 days. Post-fermentative maceration was concluded to have a positive effect on the quality of the wine, 40–50 days being the optimum time to increase the phenolic compound concentration and antioxidant activity without negative repercussions on sensory properties.

**Table 4:** Total phenolic concentration (mg/L) of wines that have undergone a post-fermentative maceration process. * CW: control wine (without post-fermentative maceration); SW: single post-fermentative maceration; DW: double post-fermentative maceration; n.d.: not detected; -: this compound has not been analyzed during this research.

References	Rivero et al. [170]			Francesca et al. [172]						Casassa et al. [171]							
Variety	Syrah			Aglianico						Pinot Noir				Zinfadel			
Compounds (mg/L)/Treatment	CW *	SW *	DW *	Initial wine	Post-fermentation maceration					Control	1 month	6 months	Double pomace	Control	1 month	6 months	Double pomace
					Day-13	Day-20	Day-50	Day-70	Day-90								
Acylated syringic acid	-	-	-	n.d.	6.05	7.58	21.04	31.61	40.01	-	-	-	-	-	-	-	-
Caffeic acid	-	-	-	7.11	6.18	6.25	7.97	9.93	13.1	-	-	-	-	-	-	-	-
Caffeoylquinic acid	-	-	-	120.7	157.91	176.5	146.40	177.5	168.90	-	-	-	-	-	-	-	-
(+)-Catechin	34.64	23.33	36.62	37.49	58.14	59.52	119.1	93.95	156.90	-	-	-	-	-	-	-	-
Catechin gallate	-	-	-	5.78	10.50	6.78	14.17	9.71	13.17	-	-	-	-	-	-	-	-
*p*-Coumaric acid	26.15	26.17	26.13	2.29	4.55	7.87	12.69	15.41	21.84	-	-	-	-	-	-	-	-
*p*-Coumaroylquinic acid	-	-	-	30.77	55.06	63.13	50.11	55.4	57.06	-	-	-	-	-	-	-	-
Cyanidin-3-*O*-glucoside	-	-	-	-	-	-	-	-	-	0.19	0.72	0.62	0.33	0.22	1.09	0.91	0.25
Delphinidin-3-*O*-coumaroyl-glucoside	-	-	-	-	-	-	-	-	-	-	-	-	-	0.29	0.31	0.15	2.11
Delphinidin-3-*O*-glucoside	0.98	0.50	0.77	-	-	-	-	-	-	2.84	3.37	3.58	2.11	2.08	3.10	1.83	0.61
Delphinidin-3-*O*-glucoside-ethyl-catechin	-	-	-	-	-	-	-	-	-	-	-	-	-	1.47	1.35	0.75	2.44
(−)-Epicatechin	58.07	33.15	51.98	10.21	20.57	25.19	54.16	29.87	78.30	-	-	-	-	-	-	-	-
Epicatechin gallate	-	-	-	n.d.	2.92	3.34	4.42	4.78	6.89	-	-	-	-	-	-	-	-
(−)-Epigallocatechin	-	-	-	52.83	53.89	59.19	47.26	51.08	54.18	-	-	-	-	-	-	-	-
Gallic acid	61.93	67.98	71.70	7.59	26.25	34.92	79.77	117.80	126.80	-	-	-	-	-	-	-	-
(−)-Gallocatechin	-	-	-	n.d.	2.42	0.93	0.84	0.73	1.08	-	-	-	-	-	-	-	-
Hydroxycinnamic acid	-	-	-	n.d.	10.45	12.00	8.23	9.69	9.76	-	-	-	-	-	-	-	-
Isorhamnetin-3-*O*-glucoside	1.95	1.51	1.88	-	-	-	-	-	-	-	-	-	-	-	-	-	-
Kaempferol-3-*O*-glucoside	n.d.	n.d.	n.d.	-	-	-	-	-	-	-	-	-	-	-	-	-	-
Laricitrin-3-*O*-glucoside	1.26	1.03	1.15	-	-	-	-	-	-	-	-	-	-	-	-	-	-
Luteolin 7-*O*-glucoside	-	-	-	4.55	8.09	8.53	3.16	4.74	3.92	-	-	-	-	-	-	-	-
Malvidin-3-*O*-acetylglucoside	13.75	5.28	9.31	-	-	-	-	-	-	-	-	-	-	9.12	8.81	2.98	4.71
Malvidin-acetylglucoside-vinyl-catechol	-	-	-	-	-	-	-	-	-	-	-	-	-	0.59	0.42	0.16	0.27
Malvidin-acetyl-vinyl-phenol	-	-	-	-	-	-	-	-	-	0.57	1.28	0.40	1.44	0.14	0.10	0.26	0.11
Malvidin-3-caffeoyl-glucoside	-	-	-	-	-	-	-	-	-	-	-	-	-	5.29	5.00	2.27	2.20
Malvidin-3-*p*-coumaroyl-glucoside	6.57	2.09	3.87	-	-	-	-	-	-	-	-	-	-	0.92	0.66	0.38	1.32
Malvidin-3-*O*-glucoside	21.85	6.87	15.13	-	-	-	-	-	-	81.75	29.82	30.64	9.62	69.01	71.55	22.55	49.09
Malvindin-3-glucoside-ethyl-epicatechin	-	-	-	-	-	-	-	-	-	1.58	2.71	2.95	0.92	-	-	-	-
Malvindin-3-glucoside-ethyl-gallo-catechin	-	-	-	-	-	-	-	-	-	0.34	0.68	0.54	0.17	-	-	-	-
Malvidin-3-*O*-glucoside-vinyl-catechol	-	-	-	-	-	-	-	-	-	-	-	-	-	0.54	0.56	0.21	0.52
Myricetin-3-*O*-glucuronide	3.63	3.09	3.55	-	-	-	-	-	-	-	-	-	-	-	-	-	-
Peonidin-3-*O*-acetylglucoside	4.53	1.93	2.33	-	-	-	-	-	-	-	-	-	-	4.15	3.48	2.13	4.61
Peonidin-3-caffeoyl-glucoside	-	-	-	-	-	-	-	-	-	-	-	-	-	0.43	0.46	0.35	0.27
Peonidin-3-*p*-coumaroyl-glucoside	1.90	0.70	1.01	-	-	-	-	-	-	-	-	-	-	-	-	-	-
Peonidin-3-*O*-glucoside	2.87	1.05	1.83	-	-	-	-	-	-	5.72	2.09	2.14	0.31	0.86	0.89	0.28	0.61
Peonidin-3-glucoside-vinyl-catechol	-	-	-	-	-	-	-	-	-	0.52	0.17	0.05	0.24	-	-	-	-
Petunidin-3-*O*-acetylglucoside	1.49	0.62	0.75	-	-	-	-	-	-	-	-	-	-	-	-	-	-
Petunidin-3-*p*-coumaroyl-glucoside	1.25	0.43	0.48	-	-	-	-	-	-	-	-	-	-	-	-	-	-
Petunidin-3-*O*-glucoside	1.93	0.78	1.40	-	-	-	-	-	-	1.39	0.32	0.23	0.03	1.02	0.70	0.23	0.40
Procyanidin B1	3.17	2.53	3.20	-	-	-	-	-	-	-	-	-	-	-	-	-	-
Procyanidin B2	2.50	2.66	3.26	-	-	-	-	-	-	-	-	-	-	-	-	-	-
Procyanidin B2 3-*O*-gallate	1.27	1.19	1.23	-	-	-	-	-	-	-	-	-	-	-	-	-	-
Procyanidin B7	1.71	1.41	1.59	-	-	-	-	-	-	-	-	-	-	-	-	-	-
Protocatechuic acid	42.21	42.19	42.24	4.33	5.90	7.10	6.56	7.33	7.49	-	-	-	-	-	-	-	-
Pyrogallol	-	-	-	2.44	5.78	3.21	5.72	6.61	6.93	-	-	-	-	-	-	-	-
Quercetin	-	-	-	n.d.	n.d.	n.d.	n.d.	n.d.	2.02	-	-	-	-	0.37	0.47	1.02	0.79
Quercetin-3-*O*-glucoside	4.72	4.08	4.93	-	-	-	-	-	-	-	-	-	-	-	-	-	-
Quercetin-3-*O*-glucuronide	4.34	2.89	4.15	-	-	-	-	-	-	-	-	-	-	-	-	-	-
Rutin	-	-	-	2.01	4.94	n.d.	2.81	n.d.	4.91	-	-	-	-	-	-	-	-
Syringic acid	44.06	44.72	44.54	31.33	42.08	49.04	37.10	44.78	41.06	-	-	-	-	-	-	-	-
Syringetin-3-*O*-glucoside	1.31	1.20	1.52	-	-	-	-	-	-	-	-	-	-	-	-	-	-
*trans*-Resveratrol	-	-	-	0.94	2.68	4.44	5.37	6.24	9.48	-	-	-	-	-	-	-	-
Vaillinic acid	42.22	46.00	47.34	4.25	9.21	10.68	8.71	9.34	9.91	-	-	-	-	-	-	-	-
*α*-Viniferin	-	-	-	1.27	3.47	5.48	4.05	5.31	6.30	-	-	-	-	-	-	-	-
Vitisin A	-	-	-	-	-	-	-	-	-	1.39	0.84	0.82	0.50	6.22	6.09	3.45	2.90
Vitisin B	-	-	-	-	-	-	-	-	-	1.08	0.41	0.24	0.31	2.48	1.85	1.56	1.00

#### 3.2.7. Filtration

One of the final steps before bottling wine is the filtration. It is commonly used as a cleaning agent and for sterilization purposes. However, it is important to understand the influence of this stage/treatment in the final phenolic compound composition. Most wineries use membrane filters because they are generally cheaper, and easy to obtain and use. Arriagada-Carrazana et al. [173] evaluated the influence of this kind of filtration on phenolic compound composition. The authors selected Cabernet Sauvignon wine that was filtered by two kinds of membrane filters. The results showed that the filtration led to a decrease in the concentration of tannins (4.8%), anthocyanins (2.4%), and a 10% reduction in the total polyphenolic index, with important consequences for the loss of color intensity.

Vernhet et Moutounet [174] evaluated the nature of the membrane used in the retention of the phenolic compounds during filtration. They used three membranes with a similar pore size but of a different nature: two hydrophilic (polyethersulfone and polyvinylpyrrolidone) and one hydrophobic (polyvinylchloride) membrane for the filtration of Carignan Noir wine. The results proved that the hydrophilic membranes produced higher adsorption of total phenolic compounds than the hydrophobic ones, which was related to the chemical structure of phenolic compounds. Furthermore, it was observed that this adsorption was duplicated in just 24 h which means that a bad cleaning process or not being replaced in the correct moment could cause a significant loss of phenolic compounds during the filtration process. These results were in consonance with those observed by Ulbricht et al. [175], who evaluated the effect of the use of microfiltration by polypropylene membrane on white wine.

### 3.3. Wine Storage

Aging has proved to be one of the most influential factors on phenolic compound concentration, with a notable transformation/evolution of these compounds with the passing of the time. The aging process is influenced by multiple factors. In the current review we evaluate the following: chemical reaction, temperature, the barrels, and the stopper.

#### 3.3.1. Chemical Reaction

During the aging process, phenolic compounds undergo numerous chemical reactions. Some of these reactions involve anthocyanins, which are transformed into more stable oligomeric and polymeric pigments with important consequences for the color and astringency properties. Flavanols also participate in condensation reactions. For this reason, the aging process is required in some cases to achieve optimal quality. However, longer periods of aging could cause oxidation reactions with unpleasant consequences. In general, older wines have higher hue values and higher concentrations of polymeric pigments, while younger wines have higher concentrations of anthocyanins and other simple phenolic compounds. These chemical reactions depend on certain internal factors (initial concentration of phenolic and volatile compounds, additives, oxygen, pH, acidity, minerals, microorganism, etc.) and external factors (temperature, barrels, closure, humidity, luminosity) [176,177,178]. In the case of the internal factors, the variety of grape has been the subject of in-depth studies. Cassino et al. [179] observed for example that a great variability in the phenolic composition of red wines (Barbera, Nebbiolo, Ruchè and Grignolino) was achieved after 4 years of aging at optimal temperature (12 °C), whereas Agazzi et al. [180] showed that Malbec wines from different zones of Mendoza and California decreased their anthocyanin content in just 5 years, influenced 40% by the time and 20% by the region.

Monagas et al. [181] also evaluated the evolution of phenolic compounds in Tempranillo, Graciano and Cabernet Sauvignon wines during 26 months of aging in bottle. The results showed that total anthocyanins decreased during aging in bottle (43% for Tempranillo, 65% for Graciano and 66% for Cabernet Sauvignon). Increases were found in catechins and proanthocyanidins, and a decrease in low-polymerized polyphenols, suggesting condensation reactions during ageing in bottle are highly dependent on the variety studied.

#### 3.3.2. Temperature

The storage temperature has proved to be a decisive factor during the aging process. In fact, the temperature is directly related to the kinetics of the reactions that occur during aging [182]. Butzke et al. [183] evaluated the effect of high temperatures during commercial shipment on the composition and the aging process of wine. The commercial shipment led to the wines being exposed to temperatures around 24 °C for a long period of time. The results showed that wines that have suffered this treatment exhibit aging between 1 and 18 months older than the wines with conventional storage at 13 °C, revealing the important influence of temperature on the aging process and, therefore, the degradation of phenolic compounds. In addition, other authors such as Puech et al. [184] or Giuffrida de Esteban et al. [185] observed that red wines preserved around 15 °C, presented higher anthocyanins levels and showed lower oxidized and fruity aromas in comparison with temperatures around 25 °C. This has been associated with the reaction of anthocyanins with H_2_O_2_ generated during molecular oxygen activation, which is favored by higher temperatures. In addition, the tannin level was higher at temperatures around 15 °C than at 25 °C. It has been estimated that the higher temperatures with the low pH of wine accelerate the loss of high molecular weight tannins.

#### 3.3.3. Barrels

The type of wood barrels producers usually choose is influenced by empirical and economic factors. Nevertheless, Frangipane et al. [186] proved the influence of the kind of wood used on the aging process. The authors stored Merlot wine for 7 months in new medium-toasted oak barrels from four different French forests: Allier (A), Never (N), Tronçais (T) and Limousin (L). Significant differences were detected in phenolic concentrations according to the kind of wood used, with coefficients of variation higher than 5% in all cases. However, it was not possible to detect a kind of wood which increased all the phenolic contents when new barrels were used. In addition, in the next year, the authors repeated the same process with the same barrels. The phenolic concentration decreased during the first reuse except for vanillin and ellagic acid, whose concentrations increased. Related to the kind of wood used, no significant differences were observed when the polyphenol concentration decreased. 

The toasting process has been shown to have the greatest influence on the final wood composition because the heat causes the chemical transformation and degradation of wood, affecting lignin, polyosides, polyphenol and lipids among others. In general, wood toasting produces a decrease in astringency and an increase in aromatic substances [187]. 

Fernández de Simón et al. [188] evaluated the effect of toasting wood barrels on the final composition of Tinta del País wine. Quercus pyrenaica was the wood selected for the barrels as it is one of the most common in Spain and the most used in wineries. When three levels of toasting were evaluated (light (115–125 °C), medium (200–210 °C), and heavy (220–230 °C)), O-cresol, phenol, coniferaldehyde, sinapaldehyde and syringol were found to be the most influenced compounds from the wood, with heavy toasting producing concentrations at least three times higher than those found with the light toasting. In addition, toasting also produces the cleavage of α–β bonds of cinnamic aldehydes and their thermo-oxidation and thermo-decarboxylation, leading to the formation of dimethoxyphenyl units, such as syringol and other simple phenols. Finally, it was concluded that the heavy toasting process produced a higher evolution of phenolic compounds in wine.

In addition, Dumitriu et al. [189] studied the influence of toasting Quercus Robur oak wood barrels on Feteasca Negra wines. In this case, it was detected that the wines aged in light toast barrels were those with the highest phenolic concentration. In fact, it was detected that after 1.5 months of aging the wine exhibited 655 mg/L of total phenolic compounds for light toasting, 567 mg/L for medium, and 576 mg/L for high toasting process whereas, after 3 months of aging, the total phenolic compounds quantified were 732 mg/L for light toasting, 681 mg/L for medium, and 512 mg/L for high toasting. 

Lastly, Watrelot et al. [190] researched toasting in *Quercus Sessilis* wood barrels, more specifically its effects on the anthocyanin content of three wines. All the wines were Cabernet Sauvignon variety, two of them elaborated in Californian wineries, while the third one was produced in a Washington state winery. One wine from the Californian winery and the wine from Washington state did not show any significant effect caused by the toasting process whereas the last wine showed a significantly greater loss of all monomeric anthocyanins (except delphinidin-3-*O*-glucosides) when the high toasting process was evaluated in comparison with low toasting. This could be the result of high toasting temperatures leading to a lower ellagitannin concentration, which limits the barrier against oxidation, resulting in a higher loss of anthocyanins. Other authors such as Collins et al. [191], Gimenez Martinez et al. [192], and Cadahía et al. [187] evaluated the effect of toasting processes on Spanish, American and French wood barrels. The results showed that many of the compounds of interest in the toasted wood arising from the degradation of lignin or hemicellulose exhibited a higher volatile content than the untoasted wood. In addition, characteristic compounds of the oak aroma such as oak lactones, eugenol, vanillin and syringaldehyde increase their levels with heating, reaching their maximum content between 195–215 °C, with an important degradation at temperatures higher than those. For this reason, many authors suggest the use of medium-level toasting for wooden barrels, thus allowing for a good evolution of the phenolic compounds in them without the loss of the characteristic oak properties.

The conclusion can be drawn that the kind of wood used in barrels has a clear and important influence in the development of polyphenols on wine. However, the different kinds of wood do not have the same influence on the different polyphenols, this being highly related to their chemical nature. On the other hand, reusing barrels has been shown to result in a decrease in the polyphenol concentration in most cases.

#### 3.3.4. Stopper

The closure used during bottling is a key factor during aging since it is the only permeable membrane between the wine and the environment. This membrane regulates the amount of oxygen that enters the bottle and the migration of some volatile compounds. In this way, closures with higher permeability would favor color stabilization but promote the oxidation of phenolic and aroma compounds [193].

Multiple authors have proved that the use of cork or synthetic closures that are more permeable to oxygen than screw caps results in a more intense color and caramel and red fruit attributes, and less vegetative and animal (reductive) aromas [194,195]. Giuffrida de Esteban et al. [185] studied the influence of different stoppers on the evolution of phenolic compounds during the aging process of Malbec wine. In addition, Wirth et al. [196] studied the impact of four oxygen transfer rates (0.8, 1.9, 8.0, and 11.9 µL oxygen/bottle/day) on the phenolic composition of Grenache red wines using synthetic closures with controlled oxygen permeability. The results revealed a progressive decrease in proanthocyanidin and hydroxycinnamic acid concentrations without a clear influence of the kind of stopper used. However, the loss of free SO_2_ and favan-3-ol monomers, and the conversion of anthocyanins into other pigments increased with the oxygen rate. It was concluded that exposure to oxygen has an important influence on the oxidation of some phenolic compounds, especially anthocyanins.

Finally, Rossetti et al. [197] examined the effect of different stoppers (blend of natural cork microgranules, one-piece natural cork, agglomerated natural cork, and technical cork) on the phenolic and volatile composition of three red wines (Merlot, Lagrein red, and St. Magdalener) and one rosé (Lagrein rosé) stored in bottles for 12 months. The results showed that the Lagrein red wine closed with the “blend” stopper showed significantly lower anthocyanin glucosides, acetyl glucosides, catechin and caffeic acid in comparison with wines closed with the other stoppers. The St. Magdalener wines showed a lower concentration of anthocyanin glucosides and acetyl-glucosides after six months of storage when blend stoppers were used. On the contrary, in the case of Merlot wines, the anthocyanin acetyl-glucosides were considerably higher when blend stoppers were used compared to the other kinds. The Lagrein rosé wine exhibited a higher concentration of *p*-coumaric acid in the bottles closed with the blend stopper. 

It can be concluded that the effect of the different kinds of stoppers is highly influenced by the kind of wine studied. 

## 4. Extraction Methods

Phenolic compounds are quantified in the wine with different purposes, among others, because they are considered quality markers. As explained above, multiple variables affect the phenolic content in grapes, and, therefore, in wine. 

Regarding phenolic compound extraction methods, many authors did not use any kind of sample pretreatment to extract phenolic compounds from wine, only dilution in some cases. Regarding the grape analysis, they are usually crushed manually to obtain the must. Frequently, the must is filtered and different pre-treatments are employed to reduce the sugar concentration, which could interfere in the analytical technique, this being the usual way samples are prepared.

In other cases, extraction techniques have been suggested with the aim of reducing the matrix interferences. Solid extraction and liquid extraction are the most common techniques for the pretreatment of the must and wine before the analysis due to the multiple advantages associated with them. They are simple to use, do not need expertise, solvents are not always required, and the extraction time is usually short. However, the drawbacks of this methodology include low selectivity, often a small enrichment factor of the analyte, possible loss of volatile compounds by opened containers and the possible loss of intensity in the sample by interaction with solvent molecules. In addition, a filtration step and repeated extraction may be required.

Yang et al. [198] utilized a dispersive liquid–liquid microextraction followed by high pressured liquid chromatography for the quantification of phenolic compounds in red, rose, and white wines (Method 1, Table 5). The extraction recoveries ranged from 76.56% to 137.74%, and the limits of detection and quantifications were between 0.001 to 0.433 mg/L. Catechin was the dominant phenolic compound in all the wines, ranging from 3.02 to 72.89 mg/L. The results showed that dispersive liquid–liquid microextraction can be considered a fast and efficient method for the extraction of phenolic compounds in wine. 

Viñas et al. [199] proposed the use of solid-phase microextraction combined with GC-MS for the detection and quantification of phenolic compounds in ten samples of different types of wine (red, rosé, and white) and grapes (red and white). The methodology details can be found in Method 2, Table 5. As observed, catechin was the main phenolic compound, with concentrations from 0.05 mg/L to 0.12 mg/L. In this case, the suitability was verified of the solid-phase microextraction for the extraction and later detection and quantification of phenolic compounds in grapes and wine.

Pérez-Jimenez et al. [200] suggested the use of headspace sorptive extraction for the analysis of different compounds in commercial Spanish wines. Their results (Method 3, Table 5) proved the capacity of this technique to be used in the quantification of esters, ketones, terpenes, alcohols, lactones, norisoprenoids and phenolic compounds. These results, together with the advantages described by authors such as its ease of use, eco-friendly methodology, and fast response, make this extraction technique a good option.

On the other hand, Robles et al. [201] used ultrasound-assisted solvent extraction of porous membrane-packed liquid samples to analyse the organic acids and polyphenols in Polish wines. Extra information about the variety used was not provided by the authors. For this reason, the average concentrations for red, rosé, and white wine have been calculated (Method 4, Table 5). As previously mentioned, catechin was the most abundant phenolic compound quantified in the wine, followed by *p*-coumaric acid in the rosé wine and caffeic acid in the red and white wines. The results proved the suitability of the ultrasound-assisted technique for use in the extraction of phenolic compounds from wine.

Prazeres et al. [202] evaluated the use of microwave-assisted extraction for the quantification of phenolic compounds in wine. This methodology (Method 5, Table 5) was applied to five different *Vitis Vinifera* varieties, and gallic acid, caffeic acid, resveratrol, epicatechin, and catechin were quantified. This methodology proved its suitability to be used for the quantification of phenolic compounds in wine with good repeatability and accuracy (RSD < 5%). Microwave-assisted extraction presents important advantages such as moderate investments, lower volatile compound loss, prevention of air-borne contamination, and the absence of hazardous fumes. However, it also shows important disadvantages for use with components of wine, such as possible lipid oxidation, no high solution temperature, single-step excludes solvent addition and the vessel should be cooled to void the loss of volatile compounds.

## 5. Analytical Techniques

**Figure 3:**
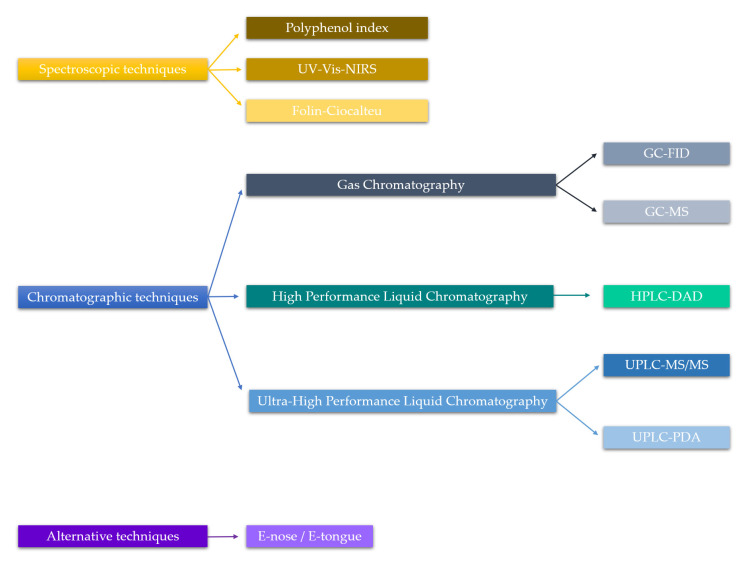
Summary diagram about the analytical techniques studied for phenolic compounds analysis on the grape, must, and wine.

Several techniques have been employed to quantify the phenolic compounds in must and/or wine. Some of these methods are based on spectroscopic techniques such as NIRs and UV absorbance (polyphenol index, Folic-Ciocalteu index). On the other hand, some authors have selected chromatographic techniques for the analysis and quantification of phenolic compounds, high-performance liquid chromatography, gas chromatography and ultra-high performance liquid chromatography being the most widely used. Finally, in recent years, eco-friendly alternatives have been suggested by other authors with the aim of covering some of the disadvantages observed with the traditional methodologies. The description of these methodologies and some examples are described below. A summary diagram of the analytical techniques most commonly used for the identification and quantification of polyphenols in grape, must and wine can be found in Figure 3.

### 5.1. Spectroscopic Techniques

The polyphenol index (I_280_) is based on the phenolic concentration and the absorbance under UV light, specifically at 280 nm. This method is based on the fact that all phenolic compounds have some absorbance at this wavelength [203]. For this methodology, wine is first diluted with deionized water (1:50) and then the absorbance is measured directly. This method has been extensively used due to its simplicity and low cost [204]. However, one problem is that each class of phenolic compounds has a different absorptivity (extinction coefficient). As a consequence, the results cannot be related with a specific standard and they cannot be directly related in absorbance units or arbitrary units. In addition, other compounds such as amino acids and proteins absorb at 280 nm as well, causing signal interferences and therefore overestimations in the phenolic quantification [205]. Thus, this method is less sensitive and more unspecific. 

Martelo-Vidal et al. [206] employed UV-Vis-NIRS technology to measure the total phenolic compounds of thirty-nine red wines. Samples were analyzed in a spectrophotometer in transmittance mode at 2 nm intervals in UV-Vis-NIRS regions (190–2500 nm). No kind of pretreatment was required, the samples were only equilibrated at 33 °C for 10 min and filtered by 0.45 µm filter before scanning. Cell quartz of 1 mm path length was used to scan the samples. The total phenolic compounds of those wines were quantified by the authors and compared with the HPLC methodology used as a reference. The results showed the possibility of using this methodology for the quantification of total phenolic compounds. It presents several advantages such as being simple, cheap and fast. However, many other compounds that are present in wine such as proteins or amino acids produce a signal in UV-Vis-NIRS regions, which is the reason why this technique cannot be considered selective. 

The Folin–Ciocalteu index (FCI) is a colorimetric assay based on the inhibition of the oxidation of the Folin–Ciocalteu reagent, which is a mixture of phosphomolybdate and phosphortungstate. This methodology is used for measuring the concentration of total phenolic compounds. This technique has been successfully applied by several authors and it is current used in industry and quality laboratories [204,207,208]. Garcia-Hernández et al. [209] used the total polyphenol index and the Folin–Ciocalteu methodology to quantify the total phenolic compounds present in wine samples (Method 1, Table 6). To this end, wine was diluted 1:5 in ultrapure water. A total of 0.1 mL volume of wine sample, 5 mL of distilled water, 0.5 mL of Folin–Ciocalteu reagent and 2 mL of 20% *w*/*w* sodium carbonate solution were added to a 10 mL calibrated flask, diluted to volume with distilled water and left for 30 min under darkness at room temperature (25 °C). Finally, the absorbance was measured at 765 nm. The total content was expressed in mg/L equivalents of gallic acid, which is used as a standard reference. The authors selected three red wine varieties (Syrah, Graciano and Tempranillo) with the aim of observing possible differences between them. In addition, from the Tempranillo variety two denominations of origin (Ribera and Toro) and three aging states (Joven, Crianza, and Reserva) were selected to evaluate the influence of both parameters as well. The FCI was able to differentiate the above samples.

Sandoval-Ventura et al. [210] successfully evaluated the use of the Folin–Ciocalteu reaction method (Method 2, Table 6) for the quantification of total polyphenols in three different *Vitis Vinifera* varieties. 

In general, the Folin–Ciocalteu methodology is considered to present important advantages for the quantification of total polyphenols in wine such as portable devices, not requiring a complex strategy, high sensitivity, and good reproducibility and repeatability. However, some important disadvantages have also been highlighted such as long reaction times (around 30 min) and low specificity [148,211]. It measures the total reducing capacity of a sample, not just the level of phenolic compounds. 

As observed, spectroscopic technologies are a good option for quantifying the total phenolic compounds in wines, presenting several advantages such as simplicity, low cost and relatively short times of analysis.

### 5.2. Chromatographic Techniques

#### 5.2.1. Gas Chromatography

Gas chromatography (GC) is extensively used for the analysis of phenolic compounds in must and wine. This technique has proved it can be successfully used in the separation of analytes from complex matrices such as wine or must. In addition, it has been widely used by many authors in different matrixes (including grape, must, and wine), resulting in there being a significant amount of literature and knowledge about the technique. Gas chromatography is an analytical technique for the analysis of volatile compounds. However, some phenolic compounds such as heterosides, anthocyanidin, or biflavonoids are not volatiles due to their high molecular weight. For this reason, a previous derivatization step is usually required. This technique has been coupled with a variety of detectors for the identification of interesting compounds such as phenolic compounds. The choice detector is made by the authors based on the nature of the sample (grape, must or wine), the purpose of the research, and the sensitivity required.

- GC-FID

Gas chromatography coupled to a flame ionization detector (FID) has been previously used by different authors for the identification/quantification of aroma-active compounds in food products, including wine. Prazeres et al. [202] suggested the use of hexamethyldisilazane as a silanizing agent in microwave-assisted derivatization for the quantification of the phenolic compounds in two Chilean wines (Carménère variety) and seven Brazilian wines (Cabernet Sauvignon, Syrah, Merlot, and Tannat varieties). For the analysis, the authors used a GC column that was subjected to a heat ramp from 110 °C to 340 °C in a total of 46 min, ensuring the separation of the compounds. After that, the compounds crossed to the FID detector at 320 °C and hydrogen gas and synthetic air were used at flow rates of 40 mL/min and 400 mL/min respectively for the ionization.

Sánchez-Gómez et al. [212] also studied the influence of oak wood in barrels on the final volatile composition of wines. The authors used a GC-FID technique with a heat ramp as well and the identification of the compounds was performed by comparison with standards. This technique has been used by several authors [213,214] for the analysis of must and wine. The results have proved its suitability, accuracy and repeatability. This technique has limitations, however, as it is not capable of identifying and quantifying all phenolic compounds; it is only suitable for the analysis of volatile compounds, which is of great interest for the analysis of wine aroma.

- GC-MS

Mass spectrometry (MS) is one of the main detectors coupled to gas chromatography due to its high sensitivity, accuracy and repeatability, and because there is a large amount of previous knowledge about this technique.

Robles et al. [201] used an ultrasound-assisted extraction with porous membrane for the analysis of phenolic compounds in Polish wine, using a GC-MS system for their quantification. In fact, the authors selected a GC system coupled to an electron ionization ion source and a single quadrupole MS. Full-scan acquisition was performed with the mass detection range set at *m/z* 400–600. A summary of this methodology and the phenolic compounds quantified could be found in Method 3, Table 6. Viñas et al. [199] also quantified five phenolic compounds (*trans*-resveratrol, *cis*-resveratrol, piceatannol, catechin, and epicatechin) in red, rosé and white wines, and in red and white grapes. With this aim, the authors employed a solid-phase microextraction as the pre-treatment followed by the analysis by GC-MS for the quantification. In addition, Pérez-Jiménez et al. [200] evaluated the total phenolic compounds and volatile components in six *Vitis Vinifera* varieties (Verdejo, Tempranillo, Graciano, Albariño, Garnacha, Godello) with the aim of detecting differences according to the genetic factor. The authors used a headspace technique as a pre-concentration step followed by an analysis by GC-MS.

Finally, Tashakkori et al. [215] used a gas chromatograph coupled to a single quadrupole mass spectrometer for the analysis of phenolic compounds in must. 

#### 5.2.2. High Performance Liquid Chromatography

High performance liquid chromatography (HPLC) has been used by various authors to measure the phenolic concentrations in wine samples due to the advantages associated with this technique such as reliability, high sensitivity, robustness and the capacity to provide good results in complex matrices. Perestrelo et al. [216] (Method 4, Table 6) suggested the use of an HPLC system coupled to a diode array detector (DAD) at 280, 330, and 520 nm. The mobile phases selected were based on two mixtures of water/formic acid/acetonitrile, and the total time of analysis was 73 min. Polyphenols were identified by comparison with retention times of standards and with UV-Vis spectra, while quantification was performed using calibration curves from standards. The authors used this methodology with the aim of measuring the polyphenol content of Verdelho wines of different vintages. It was observed that the caftaric and coutaric acid concentrations were the most abundant compounds that be considered as “marker compounds” for the differentiation of young wines. 

Carneiro et al. [217] used the HPLC technique coupled to a Dionex MWD-300 detector fixed at 254 nm for the quantification of vanillic acid, syringic acid, ellagic acid, quercetin and melatonin (a non-phenolic compound) in 35 wine samples (9 samples from the São Francisco Valley (Brazil) and 26 samples of wine from Valle de Uco, Luján de Cuyo, Mendoza and Neuquén (Argentina). The results showed that quercetin was the predominant phenolic compound found in Argentine wines. In addition, the vanillic acid and ellagic acid contents were higher in most of the Brazilian wines. On the other hand, the syringic acid concentration was lower in the Brazilian wines than in Argentine ones. Based on the results obtained and the use of chemometric tools, it was concluded that it is possible to discriminate the origin of wines according to their phenolic composition.

Other previously mentioned researchers used the HPLC technique for the quantification of phenolic compounds with different purposes. This is the case of Frangipane et al. [186], who used the method suggested by Moutounet et al. (1989) [218] with some modifications (Method 5, Table 6). This method was used to successfully measure the polyphenol concentration in wine stored in barrels of different oak wood samples allowing the discrimination according to the kind of wood used. Gordillo et al. used the HPLC-DAD technique to confirm the possibility of using vegetable origin fining agents instead of animal origin ones, thus avoiding the disadvantages associated with them. The authors used HPLC coupled to DAD detector set at 525 nm (anthocyanins), 280 nm (benzoic acids and monomeric flavanols), 320 nm (hydroxycinnamic acids and their tartaric esters) and 360 nm (flavonols) for the quantification of phenolic compounds in treated wines.

#### 5.2.3. Ultra-High Performance Liquid Chromatography

During the last few years, many authors have used ultra-high performance liquid chromatography (UPLC) to quantify the phenolic compounds in must and wine. The increased pressure in comparison with traditional liquid chromatography makes it possible to shorten analysis times and enhance the compound separation without losing the advantages associated with the liquid chromatography technology. 

Lukić et al. [219] suggested the use of UPLC-MS/MS (Method 6, Table 6) for the quantification of 58 phenolic compounds in 173 wines (4 red and 6 white varieties). To this end, a UPLC system with a reverse phase column was employed using a gradient of 15 min. Finally, a Xevo TQ MS system with electrospray positive ionization mode was selected for the quantification of the phenolic compounds. The results proved that the contents of many phenolic compounds could be used as varietal markers with a differentiation efficacy of 95% in all cases. In particular, peonidin 3-(6″-acetyl)-glucoside and taxifolin were specific markers for red varieties whereas *cis*-piceid was a marker for white wines.

Del-Castillo-Alonso et al. [81] tested the use of an ultraviolet-B radiation (UV-B) supplement on Tempranillo grape skins and its influence on the phenolic and volatile compounds in the resulting wines. For the evaluation of this effect, the author selected UPLC coupled to a micrOTOF II high-resolution mass spectrometer for the quantification of phenolic compounds. The UV detector module was used at 520 nm for anthocyanins and set at 324 nm for other compounds. The results confirmed the influence of the ultraviolet-B radiation on most of the phenolic compounds, with an increase in several glycosylated flavonols (quercetins, kaempferols, myricetins, isorhamnetins) in grape skins and consequently, in wine. Certain phenolic acids and flavanols were also increased by UV-B supplementation, as was antioxidant capacity. Conversely, stilbenes and anthocyanins were the phenolic compounds showing the most diffuse responses, a deeper study of this influence being required.

Wojdyło et al. [126] (Method 7, Table 6) studied the influence of three pretreatment maceration techniques (microwave, thermo-maceration, and enzymatic treatment) on the phenolic composition of Dornfelder cv. must/wine. The authors used UPLC coupled to a PDA detector. The results proved that the thermo-maceration and microwave pre-treatments produced higher phenolic compositions in the wines after maturation, the wines treated with microwaves showing the highest content.

Lastly, Gil et al. [159] studied the effect of polyvinylpolypyrrolidone treatment as a fining agent on rosés wines. The influence of this compound on the phenolic compounds in wine was performed with a UPLC system connected to a triple quadrupole mass spectrometer equipped with an electrospray ionization source (ESI) operating in switching positive and negative mode. A clear effect on color and polyphenol adsorption was observed and the values increased with the PVPP concentration used. 

### 5.3. Alternative Techniques

The electronic nose and tongue have been proposed in recent years as an alternative to the traditional methodologies for the quantification of the phenolic compounds in wine due to the many advantages it presents. Electronic techniques present high precision and sensibility and many of the methodologies based on their use neither require solvents nor generate residues. In addition, they use little energy. 

Cetó et al. [211] suggested the use of a bioelectronic nose for the analysis of polyphenol compounds in a total set of 29 wine samples of different varieties and origins. The analysis was performed at room temperature (25 °C) under quiescent conditions with a multichannel electrode. Four composite working electrodes, a double junction electrode Ag/AgCl and a platinum-based electrode were selected for the creation of the voltametric cell used. Spectrophotometric measurements were performed with a Spectronic Helios Epsilon spectrophotometer. For the electrode fabrication, EpoTek H77 resin and its corresponding hardening compounds were mixed in a ratio of 20:3 (*w*/*w*). Next, the four electrodes were prepared by adding 15% of graphite (*w*/*w*) and 2% of either the enzyme (tyrosinase or laccase) or the modifier (copper nanoparticles) before hardening. Finally, the biocomposite was manually homogenized for 60 min and allowed to harden for 7 days at 40 °C. The electrodes were immersed 3–5 times in buffer solution before the analysis. The potential was cycled between −0.4 V and +0.8 V vs. Ag/AgCl with a scan rate of 100 mV s-1 and a step potential of 9 mV. The samples were measured directly without any pretreatment. The BioElectronic Tongue based on voltametric enzyme-modified biosensors made it possible to quantify the total polyphenol content of the selected wines and to discriminate individual polyphenolic compounds by means of statistical techniques. 

Rudnitskaya et al. [220] studied the possibility of using an electronic tongue for the quantification of the organic acids and phenolic compounds in Madeira wines (Method 8, Table 6). The results were compared with those obtained by a traditional methodology (HPLC). The electronic tongue was made using a multisensor system comprising 26 potentiometric chemical sensors: plasticized PVC sensors displaying sensitivity to organic anions and phenolic compounds and to organic cations, chalcogenide glass sensors displaying redox response and a conventional glass pH electrode. Responses of the sensor array were measured vs. a conventional Ag/AgCl reference electrode. Wines were analyzed using a custom-made high input impedance multichannel voltmeter. The samples were diluted twice with distilled water to reduce the matrix interferences from lipophilic compounds. The sensors were conditioned for 10 min in red table wine and washed until measurements were made. The measurement time in wine was 8 min.

It is evident that many and varied analytical techniques exist for the detection and quantification of phenolic compounds in must and wine. However, a comparative could not be performed because the same samples were not analyzed by the different methodologies, so the different concentrations shown in Table 6 could be due to external factors not related to the techniques. Table 6 presents a summary of the advantages and disadvantages associated with the mentioned techniques, factors that should be taken into account prior to making a decision about which to use. In addition, a summary diagram about the different analytical techniques selected during this section can be found in Figure 3.

**Table 6:** Summary of techniques and methods applied for the quantification of phenolic compounds on wine.

Technique	Method	Phenolic Concentration (mg/L)	References
Spectroscopic techniques—Folin Ciocalteu methodology	**Method 1:** For the analysis of total polyphenol contents, wine samples were diluted with ultrapure water (1:100) and the absorbance was measured at 280 nm using a spectrophotometer. The value of I_280_ was calculated as the absorbance × 100. For the determination of Folin–Ciocalteu Index (FCI). Wine was diluted 1:5 in ultrapure water. Then, 0.1 mL volume of wine sample, 5 mL of distilled water, 0.5 mL of Folin–Ciocalteu reagent, and 2 mL of 20% *w*/*w* sodium carbonate solution were placed in a 10 mL calibrated flask, diluted to volume with distilled water and allowed to stand for 30 min before measuring the absorbance at 765 nm.	**Total polyphenol index:** Joven (D.O. Ribera) (67), Crianza (D.O. Ribera) (64), Gran Reserva (D.O. Ribera) (56), Joven (D.O. Toro) (82), Crianza (D.O. Toro) (64), Reserva (D.O. Toro) (58), Reserva (D.O. Rioja) “Syrah” (57), and Reserva (D.O. Rioja) “Coupage” (59). **Folin–Ciocalteu Index:** Joven (D.O. Ribera) (73), Crianza (D.O. Ribera) (69), Gran Reserva (D.O. Ribera) (61), Joven (D.O. Toro) (81), Crianza (D.O. Toro) (70), Reserva (D.O. Toro) (65), Reserva (D.O. Rioja) “Syrah” (57), and Reserva (D.O. Rioja) “Coupage” (64).	[209]
	**Method 2:** The microchip was designed containing three inlets: two for reagents (Folin–Ciocalteu and NaOH) and a third one to inject the sample. Two optical fibers using guides located parallel or perpendicular to the flow outlet were inserted into the microchip. In addition, a 6 V and 10 W halogen lamp with an optical path length of 7 mm between optical fibers was used. The microchannel width ranged from 70 to 120 lm in different microchips.	**Total polyphenols concentration:** Chenin blanc (2.14), Chardonnay (1.89), and Sauvignon blanc (2.56).	[210]
GC/MS	**Method 3**: Injection port at 250 °C. Capillary column (30 m × 0.25 mm, 0.25 µm) with helium at a constant flow of 1 mL/min. Oven temperature at 70 °C for 1 min, then increased to 280 °C at 10 °C/min and maintained for 5 min. Total time of separation: 27 min. The temperatures of the MS transfer line, ion source, and detector were fixed at 300, 230 and 150 °C. The MS was operated in positive mode (electron energy 70 eV). Full-scan acquisition was performed with mass detection range set at *m*/*z* 400–600.	**Red:** protocatechuic acid (1.08), *p*-coumaric acid (0.96), gallic acid (0.99), ferulic acid (1.03), caffeic acid (1.01), sinapic acid (1.07), pterostilbene (1.02), resveratrol (1.15), (+)-catechin (1.07). **White:** protocatechuic acid (1.12), *p*-coumaric acid (1.08), gallic acid (0.85), ferulic acid (0.98), caffeic acid (1.04), sinapic acid (1.05), pterostilbene (1.01), resveratrol (1.00), (+)-catechin (0.95). **Rosé:** protocatechuic acid (1.09), *p*-coumaric acid (0.90), gallic acid (1.03), ferulic acid (1.00), caffeic acid (0.99), sinapic acid (1.04), pterostilbene (0.97), resveratrol (0.96), (+)-catechin (1.03).	[201]
HPLC	**Method 4:** Reversed-phase 80 Å LC Column (250 × 4.6 mm, 4 μm) at 30 °C. Mobile phase water/formic acid/acetonitrile A (87:10:3) and B (40:10:50). Gradient programme: from 6 to 20% B (20 min), from 20 to 40% B (15 min), from 40 to 60% B (5 min), from 60 to 90% B (5 min), isocratic 90 B (5 min), from 90 to 6% B (0.5 min), isocratic 6% B (22.5 min). Flow rate 500 μL/min. Total analysis 73 min. Chromatograms were recorded at 280, 330, and 520 nm.	**Harvest 2010:** gallic acid (0.81), protocatechuic acid (6.02), caftaric acid (25.40), coutaric acid (12.60), caffeic acid (2.58), *p*-coumaric acid (2.01), ferulic acid (0.53), sinapic acid (0.18), procyanidin B1 (1.25), catechin (7.04), procyanidin B2 (3.39), epigallocatechin gallate (2.78), epicatechin (30.90), gallocatechin gallate (11.40), epicatechin gallate (3.70), catechin gallate (1.30), procyanidin A2 (1.84), quercetin-3-*O*-galactoside (0.53), quercetine-3-*O*-glucoside (0.87), kaempferol-3-*O*-rutinoside (3.68), and myricetin (1.58). **Harvest 2011:** gallic acid (0.14), protocatechuic acid (9.33), caftaric acid (23.90), coutaric acid (7.03), caffeic acid (2.92), *p*-coumaric acid (0.46), ferulic acid (0.52), sinapic acid (0.04), procyanidin B1 (1.23), catechin (3.92), procyanidin B2 (4.01), epigallocatechin gallate (1.25), epicatechin (61.60), gallocatechin gallate (12.70), epicatechin gallate (2.91), catechin gallate (1.37), procyanidin A2 (4.66), quercetin-3-*O*-galactoside (0.64), quercetine-3-*O*-glucoside (0.62), kaempferol-3-*O*-rutinoside (4.13), and myricetin (1.67). **Harvest 2012:** gallic acid (0.40), protocatechuic acid (7.00), caftaric acid (13.80), coutaric acid (4.88), caffeic acid (2.77), *p*-coumaric acid (2.82), ferulic acid (0.31), sinapic acid (0.03), procyanidin B1 (1.63), catechin (6.31), procyanidin B2 (4.93), epigallocatechin gallate (1.22), epicatechin (20.40), gallocatechin gallate (12.20), epicatechin gallate (3.53), catechin gallate (2.08), procyanidin A2 (1.79), quercetin-3-*O*-galactoside (0.80), quercetine-3-*O*-glucoside (0.53), kaempferol-3-*O*-rutinoside (1.99), myricetin (1.48), quercetin (1.64), and *trans*-piceid (0.001). **Harvest 2013:** gallic acid (27.80), protocatechuic acid (5.31), caftaric acid (45.50), coutaric acid (16.50), caffeic acid (2.21), *p*-coumaric acid (0.66), ferulic acid (0.48), sinapic acid (0.08), procyanidin B1 (1.32), catechin (8.95), procyanidin B2 (3.70), epigallocatechin gallate (1.59), epicatechin (4.09), gallocatechin gallate (9.59), epicatechin gallate (5.07), catechin gallate (1.94), procyanidin A2 (1.54), quercetin-3-*O*-galactoside (0.77), quercetine-3-*O*-glucoside (0.58), kaempferol-3-*O*-rutinoside (4.42), myricetin (1.53), *trans*-piceid (0.0145), and *cis*-piceid (0.030). **Harvest 2014:** gallic acid (14.2), protocatechuic acid (7.10), caftaric acid (35.4), coutaric acid (7.30), caffeic acid (6.70), *p*-coumaric acid (4.61), ferulic acid (0.41), sinapic acid (0.04), procyanidin B1 (1.31), catechin (12.10), procyanidin B2 (5.14), epigallocatechin gallate (1.17), epicatechin (6.40), gallocatechin gallate (4.11), epicatechin gallate (6.11), catechin gallate (1.85), procyanidin A2 (1.94), quercetin-3-*O*-galactoside (0.46), quercetine-3-*O*-glucoside (0.63), kaempferol-3-*O*-rutinoside (2.14), myricetin (1.45), *trans*-piceid (0.10), and *cis*-piceid (0.10). **Harvest 2015:** gallic acid (11.90), protocatechuic acid (3.88), caftaric acid (54.80), coutaric acid (13.30), caffeic acid (4.00), *p*-coumaric acid (1.23), ferulic acid (0.27), sinapic acid (0.15), procyanidin B1 (2.04), catechin (8.69), procyanidin B2 (3.02), epigallocatechin gallate (2.04), epicatechin (3.06), gallocatechin gallate (10.20), epicatechin gallate (7.30), catechin gallate (2.24), procyanidin A2 (1.17), quercetin-3-*O*-galactoside (1.27), quercetine-3-*O*-glucoside (0.40), kaempferol-3-*O*-rutinoside (0.36), myricetin (1.45), quercetin (1.56), *trans*-piceid (0.01), and *cis*-piceid (0.06).	[216]
	**Method 5:** First, 20 µL sample injected into HPLC-UV-Vis. 4 µm Nova Pak C18 (300 mm × 3.9 mm) column. Phase A: water-formic acid (98:2 *v*/*v*). Phase B: 70% of MeOH + 2% formic acid + 30% of solvent A. Flow: 0.8 mL/min. Gradient elution programme: 3 min at 0% B, to 10% B (7 min), to 40% B (50 min), to 60% B (20 min), to 100% B (20 min), and 10 min at 100% B.	**Allier:** HMF (4.08), furfural (1.09), vanillin (10.10), syringaldehyde (11.50), and ellagic acid (4.74). **Never:** HMF (3.53), furfural (0.80), vanillin (8.28), syringaldehyde (13.10), and ellagic acid (8.60). **Tronçais:** HMF (3.93), furfural (1.12), vanillin (7.21), syringaldehyde (11.80), and ellagic acid (3.79). **Limousin:** HMF (3.67), furfural (0.82), vanillin (9.72), syringaldehyde (15.20), and ellagic acid (5.55).	[186]
UPLC/ UPLC-MS/MS	**Method 6**: 25 mL of wine diluted 4 times with deionized water and loaded in C18-SPE cartridge (later washed with 6 mL of 0.3% aqueous perchloric acid and 10 mL of methanol). Evaporation of the eluate was at 30 °C, and reconstituted in 1 mL of methanol/water (1:1). Filtered through a 0.22 μm PTFE filter prior to injection. 2 μL were injected by an auto-sampler at 6 °C. Reverse phase Acquity HSS T3 column (1.8 μm, 100 mm × 2.1 mm) used at 40 °C. Flow of 0.4 mL/min. Mobile phase A: water + 0.1% formic acid and Mobile phase B: acetonitrile + 0.1% formic acid. The gradient was 5% B (0 min), to 20% B (3 min), constant until 4.3 min, followed by gradient to 45% B (9 min), and to 100% (11 min). Constant gradient 100% for 2 min and back to initial position in 2 min. Xevo TQ MS system with electrospray positive ionization mode. Capillary voltage at 0.5 kV Block and desolvation temperatures: 150 and 500 °C. Desolvation gas flow was 1000 L/h and cone gas flow 20 L/h.	**Teran:** delphinidin 3-*O*-glucoside (9.25), cyanidin 3-*O*-glucoside (2.02), petunidin 3-*O*-glucoside (30.90), peonidin 3-*O*-glucoside (16.16), malvidin 3-*O*-glucoside (122.10), delphinidin 3-(6″-acetyl)-glucoside (3.35), cyanidin 3-(6″-acetyl)-glucoside (1.74), petunidin 3-(6″-acetyl)-glucoside (8.79), peonidin 3-(6″-acetyl)-glucoside (6.53), malvidin 3-(6″-acetyl)-glucoside (31.22), delphinidin 3-(6″-*p*-coumaroyl)-glucoside (0.53), cyanidin 3-(6″-*p*-coumaroyl)-glucoside (0.37), petunidin 3-(6″-*p*-coumaroyl)-glucoside (0.90), peonidin 3-(6″-*p*-coumaroyl)-glucoside (1.32), malvidin 3-(6″-*p*-coumaroyl)-glucoside (11.22), *p*-hydroxybenzoic acid (0.50), vanillic acid (0.71), gallic acid (36.89), 2,5-dihydroxybenzoic acid (0.50), methyl gallate (0.04), ellagic acid (2.93), caffeic acid (1.00), ferulic acid (0.12), *trans*-caftaric acid (33.04), *trans*-fertaric acid (2.91), *trans*-coutaric acid (12.00), *trans*-piceid (2.19), *cis*-piceid (19.71), astringin (0.79), isorhapontin (0.24), pallidol (1.19) isohopeaphenol (0.92), catechin (40.05), epicatechin (18.85), epigallocatechin (2.77), gallocatechin (4.18), procyanidin B1 (69.63), procyanidin B2 + B4 (46.78), procyanidin B3 (17.00), quercetin (0.27), taxifolin (0.65), myricetin (1.78), laricitrin (1.32), and quercetin 3-rhamnoside (0.01). **Plavac mali:** delphinidin 3-*O*-glucoside (5.02), cyanidin 3-*O*-glucoside (0.94), petunidin 3-*O*-glucoside (17.40), peonidin 3-*O*-glucoside (7.47), malvidin 3-*O*-glucoside (82.47), delphinidin 3-(6″-acetyl)-glucoside (0.40), cyanidin 3-(6″-acetyl)-glucoside (0.22), petunidin 3-(6″-acetyl)-glucoside (1.31), peonidin 3-(6″-acetyl)-glucoside (1.15), malvidin 3-(6″-acetyl)-glucoside (8.04), delphinidin 3-(6″-*p*-coumaroyl)-glucoside (0.53), cyanidin 3-(6″-*p*-coumaroyl)-glucoside (0.22), petunidin 3-(6″-*p*-coumaroyl)-glucoside (0.68), peonidin 3-(6″-*p*-coumaroyl)-glucoside (0.84), malvidin 3-(6″-*p*-coumaroyl)-glucoside (10.41), *p*-hydroxybenzoic acid (0.72), vanillic acid (0.69), gallic acid (41.52), 2,5-dihydroxybenzoic acid (0.14), methyl gallate (0.03), ellagic acid (3.19), caffeic acid(1.00), ferulic acid (0.25), *trans*-caftaric acid (33.66), *trans*-fertaric acid (3.77), *trans*-coutaric acid (10.81), *trans*-piceid (1.93), *cis*-piceid (11.56), astringin (0.97), isorhapontin (0.35), pallidol (0.98), isohopeaphenol (1.28), catechin (47.69), epicatechin (15.59), epigallocatechin (3.96), gallocatechin (16.35), procyanidin B1 (90.96), procyanidin B2 + B4 (37.54), procyanidin B3 (14.38), kaempferol (0.02), quercetin (0.31), taxifolin (9.60), myricetin (1.00), laricitrin (1.21), quercetin 3-*O*-rhamnoside (0.47), and myricitrin (0.36). **Merlot:** delphinidin 3-*O*-glucoside (9.51), cyanidin 3-*O*-glucoside (1.45), petunidin 3-*O*-glucoside (25.06), peonidin 3-*O*-glucoside (9.98), malvidin 3-*O*-glucoside (92.08), delphinidin 3-(6″-acetyl)-glucoside (3.23), cyanidin 3-(6″-acetyl)-glucoside (1.40), petunidin 3-(6″-acetyl)-glucoside (6.86), peonidin 3-(6″-acetyl)-glucoside (4.91), malvidin 3-(6″-acetyl)-glucoside (24.49), delphinidin 3-(6″-*p*-coumaroyl)-glucoside (0.62), cyanidin 3-(6″-*p*-coumaroyl)-glucoside (0.42), petunidin 3-(6″-*p*-coumaroyl)-glucoside (0.81), peonidin 3-(6″-*p*-coumaroyl)-glucoside (1.27), malvidin 3-(6″-*p*-coumaroyl)-glucoside (9.96), *p*-hydroxybenzoic acid (0.42), vanillic acid (0.67), gallic acid (31.73), 2,5-dihydroxybenzoic acid (0.28), methyl gallate (0.04), ellagic acid (2.62), caffeic acid (0.47), ferulic acid (0.04), *trans*-caftaric acid (24.23), *trans*-fertaric acid (1.36), *trans*-coutaric acid (8.11), *trans*-piceid (1.93), *cis*-piceid(16.42), astringin (0.79), isorhapontin (0.17), pallidol (0.40), isohopeaphenol (0.28), catechin (41.96), epicatechin (18.18), epigallocatechin (4.72), gallocatechin (8.03), procyanidin B1 (68.16), procyanidin B2 + B4 (39.33), procyanidin B3 (39.33), kaempferol (39.33), quercetin (0.34), taxifolin (1.44), myricetin (1.44), laricitrin (1.04), quercetin 3-*O*-rhamnoside (0.03), myricitrin (0.10). **Cabernet sauvignon:** delphinidin 3-*O*-glucoside (10.01), cyanidin 3-*O*-glucoside (1.11), petunidin 3-*O*-glucoside (20.86), peonidin 3-*O*-glucoside (7.18), malvidin 3-*O*-glucoside (92.41), delphinidin 3-(6″-acetyl)-glucoside (4.17), cyanidin 3-(6″-acetyl)-glucoside (1.30), petunidin 3-(6″-acetyl)-glucoside (7.73), peonidin 3-(6″-acetyl)-glucoside (4.21), malvidin 3-(6″-acetyl)-glucoside (33.48), delphinidin 3-(6″-*p*-coumaroyl)-glucoside (0.26), cyanidin 3-(6″-*p*-coumaroyl)-glucoside (0.13), petunidin 3-(6″-*p*-coumaroyl)-glucoside (0.26), peonidin 3-(6″-*p*-coumaroyl)-glucoside (0.65), malvidin 3-(6″-*p*-coumaroyl)-glucoside (8.29), *p*-hydroxybenzoic acid (0.43), vanillic acid (0.46), gallic acid (28.84), 2,5-dihydroxybenzoic acid (0.31), methyl gallate (0.02), ellagic acid (4.20), caffeic acid (1.58), ferulic acid (0.06), *trans*-caftaric acid (33.00), *trans*-fertaric acid (1.45), *trans*-coutaric acid (13.30), *trans*-piceid (0.78), *cis*-piceid (6.63), astringin (0.36), isorhapontin (0.07), pallidol (0.23), isohopeaphenol (0.26), catechin (40.32), epicatechin (13.94), epigallocatechin (3.62), gallocatechin (10.27), procyanidin B1 (71.18), procyanidin B2 + B4 (34.63), procyanidin B3 (14.24), kaempferol (0.02), quercetin (0.48), taxifolin (1.33), myricetin (1.83), laricitrin (1.50), quercetin 3-*O*-rhamnoside (0.06), myricitrin (0.09).	[219]
	**Method 7:** The fruit was destemmed and crushed to obtain must. -Sample without any treatment: control sample. -Microwave treatment: for eight minutes (1200 W reaching 80 °C) -Thermo-maceration: the must was heated to 80 °C for eight minutes. -Enzymatic treatment: the must was treated with pectolytic enzyme- Siha Pectinase at a dose of 0.05 mg/L, for 1 h at 50 °C, and stirred every 15 min. Then, must was heated to 65 °C for 2 min to denature the enzyme. For the analysis, the samples were filtered through a 0.22 μm membrane and analyzed by means of an Acquity UPLC system with a PDA detector. An Acquity UPLC BEH C18 column (2.1 × 100 mm, 1.7 μm) at 30 °C was used. Next, 5 μL of sample was injected to the system at a flow rate of 0.43 mL/min. Mobile phase A: deionized water + 4.5% formic acid Mobile phase B: acetonitrile + 4.5% formic acid. Gradient: 1% B (0 min), to 25% B (12 min), and to 100% B (12.5 min). Back to initial conditions in 1 min.	**Must:** *Control*: anthocyanins (1.03), flavonols (0.22), phenolic acids (2.20), flavan-3-ols (26.54), and total phenolic (29.99). *Microwave*: anthocyanins (12.38), flavonols (2.19), phenolic acids (1.26), flavan-3-ols (27.59), and total phenolic (43.42). *Thermo-maceration*: anthocyanins (3.84), flavonols (2.41), phenolic acids (2.55), flavan-3-ols (23.15), and total phenolic (31.95). *Enzymatic*: anthocyanins (3.02), flavonols (0.62), phenolic acids (1.45), flavan-3-ols (28.96), and total phenolic (34.08). **Wine after fermentation:** *Control*: anthocyanins (0.38), flavonols (0.08), phenolic acids (0.41), flavan-3-ols (19.45), and total phenolic (20.32). *Microwave*: anthocyanins (1.01), flavonols (0.23), phenolic acids (0.88), flavan-3-ols (19.61), and total phenolic (21.73). *Thermo-maceration*: anthocyanins (0.71), flavonols (0.10), phenolic acids (0.63), flavan-3-ols (20.18), and total phenolic (21.62). *Enzymatic*: anthocyanins (0.44), flavonols (0.09), phenolic acids (0.41), flavan-3-ols (19.18), and total phenolic (20.12). **Wine after maturation (six months):** *Control:* anthocyanins (0.12), flavonols (0.04), phenolic acids (0.47), flavan-3-ols (19.55), and total phenolic (20.18). *Microwave*: anthocyanins (0.53), flavonols (0.11), phenolic acids (0.82), flavan-3-ols (20.22), and total phenolic (21.68). *Thermo-maceration:* anthocyanins (0.38), flavonols (0.09), phenolic acids (0.65), flavan-3-ols (19.26), and total phenolic (20.38). *Enzymatic*: anthocyanins (0.08), flavonols (0.02), phenolic acids (0.39), flavan-3-ols (19.48), and total phenolic (19.97).	[126]
Etongue	**Method 8:** 26 potentiometric chemical sensors: plasticized PVC sensors displaying sensitivity to organic anions and phenols and to organic cations; chalcogenide glass sensors displaying redox response and a conventional glass pH electrode. Responses of the sensor array were measured vs. conventional Ag/AgCl reference electrode.	**Madeira wine:** protocatechuic acid (4.66), catechin (0.76), vanillinic acid (3.23), vanillin (1.38), sinapic acid (1.32), and *trans*-resveratrol (0.23).	[220]

## 6. Conclusions

This review describes phenolic compounds including types, properties, effects on wine quality, extraction methods and techniques for their quantification. The quality-related properties of polyphenols have been widely studied for some years. More recently, numerous strategies and factors (pre, during and post alcoholic fermentation) have been evaluated to manage the phenolic content in wines. The design of a certain type of wine starts with the selection of a grape variety and its behavior under the invariable climate conditions in the growing region. In fact, the climate was found to have the most influential effect on grape composition, followed by soil and cultivar. The results showed that water available on the grapevine is a decisive parameter for the phenolic compound concentration. Autochthonous varieties are usually well adapted to the local climatic parameters. To increase the phenolic content in grapes, biostimulants are usually added to the grapevine during the grape ripening process. Vine-shoot extracts and chitosan can be proposed as the most promising biostimulants, with significant consequences for the phenolic compound content in grapes and wine. 

Once grapes have been harvested, the winemaker may act at different stages. During the pre-fermentative stage, maceration and thermovinification are recommended for young red wines, these having a significant effect on phenolic compounds, especially anthocyanins since they are easily extracted in aqueous media. Using mixed culture non-*Saccharomyces* plus *Saccharomyces* has been observed to increase the phenolic composition of final wines. Lastly, post-fermentative maceration can be suggested for wine aged in barrels since tannins are easily extracted in alcoholic media. The selection of additives, fining agents, filters, stoppers and storage conditions play an important role in the phenolic composition of wine, the choice depending on the type of wine desired. More natural and fewer chemical compounds are proposed to obtain more natural wines. For example, protein from pea or potatoes are proposed as substitutes for PVPP or gelatin.

Regarding phenolic extraction methods in wine, many authors did not use any kind of sample pretreatment to this end, only dilution in some cases. However, when high sensibility and less interference is required, other alternatives are available such as liquid–liquid microextraction, solid-phase microextraction, headspace sorptive extraction, ultrasound-assisted extraction, and the microwave-assisted technique. 

Finally, different techniques are currently used to analyse samples. They can be summarized as: spectroscopic techniques, gas chromatography (GC), high-performance liquid chromatography (HPLC), ultra-high performance liquid chromatography (UHPLC) and other alternatives such as the electronic nose and tongue. Spectroscopic techniques are the simplest, but are less selective, while UHPLC is one of the most accurate, especially when combined with a mass detector.

## Figures and Tables

**Table 5:** Phenolic compounds concentration from *Vitis Vinifera* varieties according to different extraction methodologies. *n.d: not detected.*

Technique	Methodology	Phenolic Compounds (mg/L)	Reference
Liquid–liquid microextraction	**Method 1:** 1 mL wine was mixed with 1 mL of ethyl acetate and 500 μL of acetonitrile and extracted for 10 s. The extraction was performed twice.	Total phenolic compounds: **Red wine** (48.74–196.07). **Rose wine** (31.11). **White wine** (11.18–30.39).	[198]
Solid-Phase microextraction	**Method 2:** Wine samples were prepared by mixing 8 mL of wine and 7 mL of water. The grapes were crushed for analysis and 8 g were weighed and extracted with 2 mL of ethanol using sonication for 5 min (50% amplitude). The entire extract was centrifuged at 4000 rpm for 5 min. Finally, the supernatant was passed to 15 mL vials and filled with water. For the analysis, polyacrylate fiber was put into the 15 mL sample at 25 °C for 10 min under continuous stirring. Then, the fiber was placed in the headspace of 10 μL of the derivatization reagent (bis(trimethylsilyl)trifluoroacetamide) for 15 min at 50 °C. Derivatization was used with the aim of converting the polar non-volatile compounds into volatile derivatives that could be quantified. Lastly, desorption was performed at 280 °C for 5 min and polyphenols were quantified by GC-MS.	**Red wine:***trans*-resveratrol (0.04), *cis*-resveratrol (0.03), piceatannol (0.01), catechin (0.12), and epicatechin (0.05). **Rosé wine:***trans*-resveratrol (0.01), *cis*-resveratrol (n.d.), piceatannol (n.d), catechin (0.11), and epicatechin (n.d). **White wine:***trans*-resveratrol (0.02), *cis*-resveratrol (n.d.), piceatannol (n.d.), catechin (0.11), and epicatechin (n.d.). **Red grape:***trans*-resveratrol (0.04), *cis*-resveratrol (n.d.), piceatannol (0.02), catechin (0.05), and epicatechin (0.04). **White grape:***trans*-resveratrol (0.01), *cis*-resveratrol (n.d.), piceatannol (n.d.), catechin (0.09), and epicatechin (n.d.).	[199]
Headspace sorptive extraction	**Method 3:** 1 mL of wine was mixed with 5 mL of a hydroalcoholic solution (13% ethanol and pH = 3.5), 40 μL of internal standard solution, and 1.5 g of NaCl. A preconditioned polydimethylsiloxane stir bar (Twister) was suspended in the headspace of the vial for 3.5 h at 36.5 °C. Next, it was thermally desorbed for 30 s. Splitless thermal desorption was programmed from 40 °C to 240 °C (for 5 min) at 60 °C/min. The injection was performed with a ramp of 12 °C/min from −100 °C to 240 °C and held for 5 min. The analysis was carried out by GC-MS.	Total phenolic compounds: Verdejo (176.50), Tempranillo (361.40), Graciano (1712.80), Albariño (201.50), Garnacha (295.10), and Godello (169.30).	[200]
Ultrasound-assisted extraction	**Method 4:** The membrane was filled with 60 mg of MgSO_4_ and 25 μL of wine and placed in a 4 mL glass vial before the extraction solvent was added (1 mL to ensure the membrane was submerged). The vial was immersed in an ultrasound bath for 25 min. Then, the extraction device was removed and a nitrogen stream was used to dry the extract. Next, 30 μL of the derivatization agent (N,O-bis(trimethylsilyl) trifluoroacetamide with 1% trimethyl chlorosilane) was added and the vial was vortexed for 30 s and heated for 30 min (at 35 °C) to allow the derivatization process. Finally, 170 μL of the extraction solvent was added to the vial to reconstitute the derivatized analytes and they were heated for 15 min at the same temperature. The solution was analysed by GC-MS system.	**Red wine:** protocatechuic acid (3.75), *p*-coumaric acid (8.73), gallic acid (3.18), ferulic acid (n.d.), caffeic acid (17.17), sinapic acid (0.96), pterostilbene (n.d.), resveratrol (3.22), and (+)-catechin (1696.63). **Rosé wine:** protocatechuic acid (2.37), *p*-coumaric acid (22.30), gallic acid (0.47), ferulic acid (n.d.), caffeic acid (8.54), sinapic acid (1.03), pterostilbene (n.d.), resveratrol (2.27), and (+)-catechin (38.00). **White wine:** protocatechuic acid (1.34), *p*-coumaric acid (1.32), gallic acid (0.28), ferulic acid (n.d.), caffeic acid (6.67), sinapic acid (n.d.), pterostilbene (n.d.), resveratrol (2.30), and (+)-catechin (47.92).	[201]
Microwave-assisted technique	**Method 5:** 2 mL of wine were mixed with 100 µL of hexamethyldisilazane used as a derivatizing agent and microwave-assisted extraction was used with 40% of microwave power with a nominal power of 1200 w and output of 700 w for 180 s. Lastly, the sample was centrifuged at 1200 rpm. Analysis by GC-MS.	**Cabernet Sauvignon:** gallic acid (60.59), caffeic acid (21.56), resveratrol (1.32), (−)-epicatechin (26.24), and (+)-catechin (33.14). **Syrah:** gallic acid (47.12), caffeic acid (11.54), resveratrol (2.12), (−)-epicatechin (33.67), and (+)-catechin (36.58). **Carménère:** gallic acid (24.82), caffeic acid (11.09), resveratrol (1.38), (−)-epicatechin (11.13), and (+)-catechin (14.53). **Merlot:** gallic acid (15.62), caffeic acid (16.52), resveratrol (5.17), (−)-epicatechin (16.85), and (+)-catechin (21.80). **Tannat:** gallic acid (19.28), caffeic acid (21.49), resveratrol (1.40), (−)-epicatechin (11.75), and (+)-catechin (16.76).	[202]
